# Progressive Impairment of NK Cell Cytotoxic Degranulation Is Associated With TGF-β1 Deregulation and Disease Progression in Pancreatic Cancer

**DOI:** 10.3389/fimmu.2019.01354

**Published:** 2019-06-21

**Authors:** Eunsung Jun, Ah Young Song, Ji-Wan Choi, Hyeon Ho Lee, Mi-Yeon Kim, Dae-Hyun Ko, Hyo Jeong Kang, Seong Who Kim, Yenan Bryceson, Song Cheol Kim, Hun Sik Kim

**Affiliations:** ^1^Division of Hepato-Biliary and Pancreatic Surgery, Department of Surgery, Asan Medical Center, AMIST, University of Ulsan College of Medicine, Seoul, South Korea; ^2^Department of Convergence Medicine, Asan Medical Center, Asan Institute for Life Sciences, University of Ulsan College of Medicine, Seoul, South Korea; ^3^Department of Biomedical Sciences, Asan Medical Center, University of Ulsan College of Medicine, Seoul, South Korea; ^4^Department of Laboratory Medicine, Asan Medical Center, University of Ulsan College of Medicine, Seoul, South Korea; ^5^Department of Pathology, Asan Medical Center, University of Ulsan College of Medicine, Seoul, South Korea; ^6^Stem Cell Immunomodulation Research Center, University of Ulsan College of Medicine, Seoul, South Korea; ^7^Department of Biochemistry and Molecular Biology, Asan Medical Center, University of Ulsan College of Medicine, Seoul, South Korea; ^8^Department of Medicine Huddinge, Karolinska Institutet, Stockholm, Sweden; ^9^Department of Clinical Sciences, University of Bergen, Bergen, Norway

**Keywords:** natural killer cells, pancreatic cancer, cytotoxicity, TGF-β1, prognosis

## Abstract

Natural killer (NK) cells are key effectors in cancer immunosurveillance and can be used as a prognostic biomarker in diverse cancers. Nonetheless, the role of NK cells in pancreatic cancer (PC) remains elusive, given conflicting data on their association with disease prognosis. In this study, using conventional K562 target cells and complementary engineered target cells providing defined and synergistic stimulation for NK cell activation, a correlation between impaired NK cell cytotoxic degranulation and PC progression was determined. Peripheral blood mononuclear cells (PBMCs) from 31 patients with newly diagnosed PC, 24 patients with non-malignant tumors, and 37 healthy controls were analyzed by flow cytometry. The frequency, phenotype, and effector functions of the NK cells were evaluated, and correlations between NK cell functions and disease stage and prognosis were analyzed. The results demonstrated that effector functions, but not frequency, of NK cells was progressively decreased on a per-cell basis during PC progression. Impaired cytotoxic degranulation, but not IFN-γ production, was associated with clinical features indicating disease progression, such as high serum CA19-9 and high-grade tumors. Significantly, this impairment correlated with cancer recurrence and mortality in a prospective analysis. Furthermore, the impaired cytotoxic degranulation was unrelated to NKG2D downregulation but was associated with increased circulating and tumor-associated TGF-β1 expression. Thus, NK cell cytotoxic activity was associated with PC progression and may be a favorable biomarker with predictive and prognostic value in PC.

## Introduction

Pancreatic cancer (PC) is characterized by a remarkably poor prognosis with the lowest 5 year survival rate (~7%) of all cancers (1). The incidence of PC has been steadily increasing, and it is predicted to be the second leading cause of cancer mortality in 2030 (2). Pancreatic ductal adenocarcinoma (PDAC) is the most common subtype (~95%), and is typically identified with distant metastasis, the leading cause of PDAC-related death, due to the lack of early symptoms and predictive biomarkers (3, 4). Accordingly, therapeutic options are limited for PC, with only a minority of patients (~15%) being eligible for surgical resection, the best curative treatment for non-disseminated disease. However, the outcome of early complete resection remains poor due to high recurrence rates (~80%). Furthermore, chemotherapy and targeted therapy for advanced-stage PC still have a limited impact on patient survival (5, 6). The CA19-9 serum level is the most widely used parameter to assess PC progression, but it has limited diagnostic accuracy due to its elevation in non-malignant conditions and false negative results with individuals not expressing Lewis blood group antigen (7). Thus, it is important to identify additional and valid prognostic factors of PC progression to develop effective diagnostics and therapies.

The immune system constantly guards against cancer development, whereas its dysregulation provides a permissive environment for tumor growth, recurrence, and dissemination (8). PC is associated with local as well as systemic immunosuppression, including overexpression of TGF-β and IL-10, which promote evasion of immune surveillance (9). Natural killer (NK) cells are a subset of innate lymphoid cells that constitute a first line of defense against cancer development and metastasis (10, 11), patrolling a variety of tissues and comprising about 5–15% of total peripheral blood mononuclear cells (PBMCs) (12, 13). While T cells are MHC class I-restricted, NK cell activation is generally potentiated by the lack of MHC class I expression. Using a multitude of germline-encoded, activating receptors, NK cells can efficiently recognize and eliminate a broad spectrum of tumor cells via direct cytolysis and IFN-γ production. NK cell effector function has been linked to cancer prognosis and survival (14). In a prospective longitudinal study, subjects with low cytotoxic activity of peripheral blood NK cells had higher incidences of diverse cancers than those with medium or high cytotoxic activity (15). Defective NK cell cytotoxicity is also associated with a high familial incidence of cancer (16) and has been reported in patients with various types of cancer (17, 18). Thus, circulating NK cell function is considered a surrogate marker of anti-tumor immunity, and may provide prognostic information (19, 20). Although inconsistent with clinical prognosis, an altered but discordant frequency and/or cytotoxic activity of circulating NK cells has been observed in patients with PDAC compared with healthy controls (21–25). Notably, NK cell cytotoxicity in previous studies was mostly assessed on the basis of sum, rather than per-cell, activity using PBMCs incubated with K562 target cells, which express multiple (e.g., ligands for NKG2D, DNAM-1, and NKp30) and unidentified ligands for NK cell-activating receptors (26, 27). This complicates the accurate measurement of NK cell function and studies of the underlying mechanism, due to the variable frequency (5–15%) of NK cells within PBMCs and the heterogeneous receptor-ligand interactions between NK cells and K562 cells (14). In this regards, the assessment of NK cell function via target cells providing defined and uniform stimulation for NK cell activation may complement the results obtained with K562 cells and provide an insight into the molecular mechanism of NK cell dysfunction. Moreover, NK cell production of cytokines such as IFN-γ was not systematically assessed in those studies.

NK cells exert selective cytotoxicity against tumor cells over healthy cells, which largely rely on MHC class I-specific inhibitory receptors such as killer cell Ig-like receptors (KIRs) and CD94-NKG2A (12). In addition, NK cells isolated from peripheral blood do not respond to single activating receptors, but require combined stimulation by synergistic pairs of activating receptors to elicit effective natural cytotoxicity against tumor cells (28, 29). Given a crucial involvement of NKG2D among others in PC (30–32), NKG2D in combination with 2B4 was selected to trigger effector functions of untouched resting NK cells that mediate host's intrinsic anti-tumor activity, on the basis of their high-level constitutive expression on resting NK cells, the capacity to trigger consistent and strong NK cell activation, and well-characterized cognate ligands (33, 34). Recently, immune checkpoint receptors (e.g., PD-1 and TIM-3) have emerged as important regulators of NK cell function, which are co-opted by tumor cells as a major immune escape mechanism (35, 36). The pathophysiological adaptation of these receptors including checkpoint receptors in PC remains unclear, particularly in relation to NK cell function. Here, using conventional K562 cells as well as engineered target cells that trigger NK cell-specific receptor coactivation, we measured NK cell functions on a per-cell basis and found their progressive impairment according to disease stage. Importantly, NK cell cytotoxic degranulation was associated with cancer progression, recurrence, and patient survival, highlighting its potential prognostic value in PC.

## Materials and Methods

### Study Design and Patients

Among the patients admitted to the Division of Hepato-Biliary and Pancreatic Surgery of the Asan Medical Center, 31 PC patients who agreed to the study were included, between July 2016 and June 2017. Peripheral blood samples were collected, and PBMCs and plasma were harvested within 1 h and then cryopreserved until processed as described (37). Clinical data on age, sex, body mass index (BMI), CA19-9 levels, and total white blood cell (WBC) counts (including neutrophil, lymphocyte, and monocyte numbers) were collected. Preoperative computed tomography (CT) and magnetic resonance imaging (MRI) were performed to determine tumor resectability. The diagnosis, tumor size, degree of tumor differentiation, lymph node involvement, and TNM stage were determined by pathologic examination. Patients were stratified into the early-stage group or the advanced-stage group according to criteria of the Union for International Cancer Control (8th edition). Patients were followed for at least 1 year to document survival and tumor recurrence. No patient enrolled in this study received preoperative neoadjuvant chemotherapy or radiation therapy. In addition, as controls, 37 healthy volunteers and 24 patients with pancreatic tumors that were surgically resected and confirmed pathologically as non-malignant lesions were included. Patient clinical and demographic characteristics are summarized in Tables S1–S3. The study complied with the Declaration of Helsinki, and the protocol was reviewed and approved by the Institutional Review Board (IRB) of Asan Medical Center (IRB No. 2016-0865). The study was also registered as a clinical study (NCT03665571) with the Clinical Research Information Service (available at https://clinicaltrials.gov). Written informed consent was acquired from all participants.

### Target Cells and Culture

To measure NK cell functions in the context of defined stimulation, we employed unrelated mouse target cells engineered to express defined ligands for human NK cell-activating receptors. Thus, besides classical K562 cells (ATCC), P815 cells with stable surface expression of ULBP1 (a ligand for human NKG2D) and/or CD48 (a ligand for human 2B4) were generated and used to determine the functional capacity of NK cells from patients with PC. The expression of NKG2D and 2B4 ligands on transfected P815 cells was confirmed by flow cytometry (37). K562 cells and different P815 cells were cultured in Iscove's modified Dulbecco's medium (IMDM; Cellgro) supplemented with 10% FBS and 2 mM L-glutamine. The cells were confirmed to be free of mycoplasma contamination.

### Antibodies

The following fluorochrome-conjugated monoclonal antibodies were used to evaluate NK cell function by flow cytometry: anti-CD3-PerCP (clone SK7, BD Bioscience), anti-CD56-PE (clone NCAM16.2, BD Bioscience), anti-CD107a-FITC (clone H4A3, BD Bioscience), and anti-IFN-γ-FITC (clone 25723.11, BD Bioscience). The following fluorochrome-conjugated monoclonal antibodies were used for phenotypic analysis of NK cells: anti-CD3-PerCP (clone SK7, BD Bioscience), anti-CD56-FITC (clone NCAM16.2, BD Bioscience), anti-CD16-PE (clone 3G8, BD Bioscience), anti-NKG2D-PE (clone 149810, R&D Systems), anti-2B4-PE (CD244; clone C1.7, BD Bioscience), anti-NKG2C-PE (clone 134591, R&D Systems), anti-NKp46-PE (CD335; clone 9E2, BD Bioscience), anti-NKp44-PE (CD336; clone p44-8.1, BD Bioscience), anti-NKp30-PE (CD337; clone Z25, Beckman Coulter), anti-DNAM-1-PE (CD226; clone DX11, BD Bioscience), anti-CD48-PE (clone TÜ145, BD Bioscience), anti-KIR2DL1/S1-PE (CD158a; clone EB6, Beckman Coulter), anti-KIR2DL2/L3-PE (CD158b; clone GL183, Beckman Coulter), anti-NKG2A-PE (CD159a; clone Z199, Beckman Coulter), anti-CD69-PE (clone FN50, BD Bioscience), anti-CD57-PE (clone NK-1, BD Bioscience), mouse IgM PE isotype control (clone G155-228, BD Bioscience), anti-TIGIT-PE (clone MBSA43, eBioscience), anti-CD96-PE (clone NK92.39, Biolegend), anti-TIM-3-PE (clone 344823, R&D Systems), rat IgG2A PE isotype control (clone 54447, R&D Systems), and anti-PD-1-PE (clone EH12.1, BD Bioscience). Human Fc receptor binding inhibitor was from eBioscience/ThermoFisher Scientific.

### NK Cell Cytotoxic Degranulation and Intracellular IFN-γ Staining

Cryopreserved PBMCs including all study group samples were thawed in a single day and suspended in complete RPMI medium [RPMI-1640 medium supplemented with 10% fetal bovine serum (FBS), 2 mM L-glutamine, 100 U/ml penicillin, and 100 μg/ml streptomycin] in the presence of 50 U/ml DNase I (Roche) to remove DNA released by the dead cells and to prevent PBMC clumping. The cells were washed twice with complete RPMI medium, resuspended in the same medium at ~4 ×10^6^ cells/ml, and rested overnight at 37°C.

NK cell degranulation was determined by the cell surface expression of CD107a, as described (37, 38). Briefly, PBMCs (2 ×10^5^ cells) were mixed with an equal number of K562 cells or P815 cells stably expressing ULBP1 and/or CD48 and incubated for 2 h at 37°C. The cell pellets were resuspended in FACS buffer (PBS with 2% FBS) and stained with anti-CD3-PerCP, anti-CD56-PE, and anti-CD107a-FITC for 30 min in the dark at 4°C. Lymphocytes were gated by forward and side scatter (FSC and SSC) characteristics, and the CD107a expression on CD3-CD56+ NK cells was analyzed by flow cytometry using FlowJo software (Tree Star). NK cell cytotoxicity against K562 and P815-ULBP1+CD48 cells were determined by a Europium assay as described (29).

PBMCs (2 ×10^5^ cells) were stimulated with an equal number of K562 cells or P815 cells stably expressing ULBP1 and/or CD48 for 1 h at 37°C. Then, brefeldin A (GolgiPlug; BD Biosciences) was added, and the samples were incubated for an additional 5 h, for a total of 6 h. The cells were first stained with anti-CD3-PerCP and anti-CD56-PE antibodies for 30 min in the dark at 4°C. The PBMCs were then washed twice with FACS buffer and incubated in BD Cytofix/Cytoperm solution (BD Biosciences) for 20 min in the dark at 4°C. The cells were then washed twice with BD Perm/Wash buffer (BD Biosciences), stained with anti-IFN-γ-FITC for 30 min in the dark at 4°C, washed again, and analyzed by flow cytometry gated on CD3-CD56+ NK cells.

### Immunohistochemistry

Tumors were fixed in 10% formalin for 24 h and embedded in paraffin. The tumor tissues were sectioned at an 8 μm thickness. After deparaffinization and antigenic retrieval, the slides were labeled with a monoclonal antibody against TGF-β1 (sc-130348, 1:200, Santa Cruz Biotechnology). Labeling was detected using the avidin-biotin complex staining method. Two board-certified pathologists specializing in PC reviewed the slides and scored the degree of TGF-β1 expression from 0 to 3 (negative to high).

### Plasma Cytokine Analysis

Plasma separated from patient blood was stored at −70°C until analysis. Magnetic Luminex assays (R&D Systems, Minneapolis, MN, USA) were used to analyze 10 different cytokines (TNF-α, TGF-β1, TGF-β2, TGF-β3, IL-4, IL-5, IL-6, IL-10, and IL-13). Briefly, the patient plasma samples were incubated for 2 h with anti-cytokine antibody-coated beads. The median fluorescence intensity was determined on a Luminex instrument (Luminex, Austin, TX, USA), and the plasma concentrations of each cytokine were calculated by comparison with a standard curve.

### Statistical Analysis

All data were analyzed using GraphPad Prism v. 5.00 (GraphPad Software). Two groups were compared using non-parametric Mann-Whitney *U*-tests and Fisher's exact tests (two-tailed). Correlations between two parameters were evaluated using the non-parametric Spearman's rank correlation test. Comparisons of three or more groups were analyzed by Kruskal-Wallis test. Dunn's corrections were performed for multiple comparisons. Multivariate analysis was used to compare clinicopathological characteristics and NK cell activity of the patients included in this study. In addition, we used logistic regression analysis for categorical data and linear regression analysis for numerical data. Statistical significance was defined as *P* < 0.05, and the degree of significance is indicated as follows: ^*^P < 0.05, ^**^P < 0.01, and ^***^P < 0.001.

## Results

### Synergistic Receptor Coactivation by NKG2D and 2B4 Is Selective to NK Cells

To test NK cell selectivity of the receptor coactivation, PBMCs from healthy controls (HCs) were incubated with P815 cells expressing ULBP1 (a ligand for human NKG2D) and/or CD48 (a ligand for human 2B4). NK cell degranulation was measured by the surface appearance of CD107a (LAMP-1) on gated NK cells, which correlates with target cell lysis (39). Consistent with previous reports (37, 38), stimulation with ULBP1 or CD48 alone induced little degranulation, whereas receptor coactivation with ULBP1 and CD48 resulted in synergistic degranulation in the CD3-CD56+ NK cell population (Figures S1A,B). Likewise, synergistic NK cell production of IFN-γ following stimulation with NKG2D and 2B4 (P815-ULBP1+CD48) was observed (Figure S1C). These results validate the use of P815-ULBP1+CD48 target cells as a valuable tool to measure NK cell functions in the context of defined stimulation.

### NK Cell Functions in Patients With PC Are Impaired

**Figure 1:**
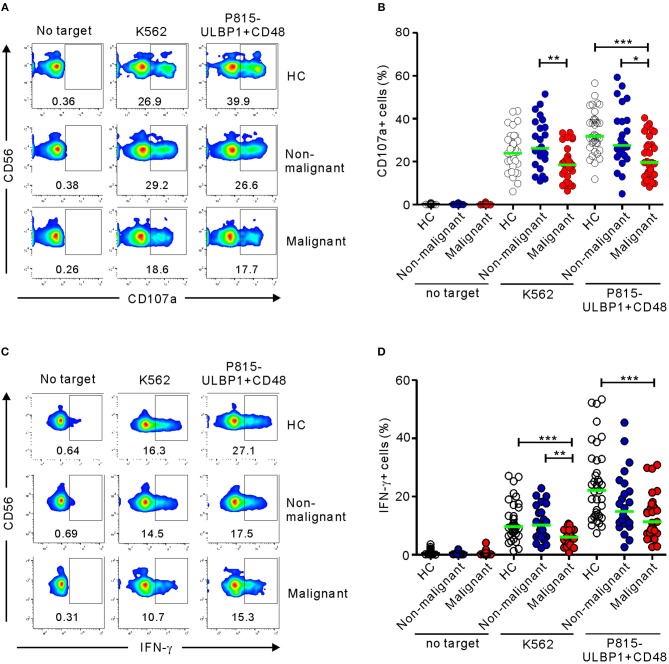
Patients with pancreatic cancer (PC) have impaired NK cell effector functions. PBMCs from the healthy control (HC) group (*n* = 37), the non-malignant group (*n* = 24), and the malignant group (*n* = 31) were incubated with either K562 cells or P815-ULBP1+CD48 cells, which activate NK cells via NKG2D and 2B4. **(A,B)** Degranulation of NK cells, as measured by cell surface expression of CD107a. **(C,D)** Cytokine production by NK cells, as measured by intracellular expression of IFN-γ. **(A,C)** Representative FACS profiles showing the percentages of CD107a+ **(A)** and IFN-γ+ NK cells **(C)**. **(B,D)** Summary graphs showing the expression of CD107a **(B)** and IFN-γ **(D)** by NK cells. Horizontal bars (green) indicate the medians. Statistical differences between the groups were evaluated with the non-parametric Kruskal-Wallis test. *P < 0.05, **P < 0.01, and ***P < 0.001.

Using both K562 cells and P815-ULBP1+CD48 cells as targets, the effector functions of CD3-CD56+ NK cells from patients with PC (malignant group) were compared with those of HCs (Tables S1–S4). NK cells from patients with non-malignant tumors (non-malignant group) were also used, to investigate whether NK cell functions are modulated in precancerous conditions. NK cells from the malignant group exhibited a significant decrease in cytotoxic degranulation compared with those from HCs in response to P815-ULBP1+CD48 cells but not K562 cells (*P* < 0.001) and those from the non-malignant group in response to both target cells (*P* < 0.01 against K562; *P* < 0.05 against P815-ULBP1+CD48) (Figures 1A,B). In support, NK cell-mediated lysis of these target cells, significantly P815-ULBP1+CD48 cells, was impaired in the malignant group compared with HCs (Figure S2). Next, the capacity of NK cells to produce IFN-γ was assessed. As observed with NK cell degranulation, NK cells from the malignant group produced significantly less IFN-γ than those from HCs against both target cells (*P* < 0.001) and those from the non-malignant group against K562 cells (*P* < 0.01) (Figures 1C,D). Thus, on a per-cell basis, NK cells of the malignant group were clearly impaired in their ability to exert cytotoxic degranulation and produce IFN-γ.

### NK Cell Dysfunction Correlates With Clinical Stage and Parameters of PC

**Figure 2:**
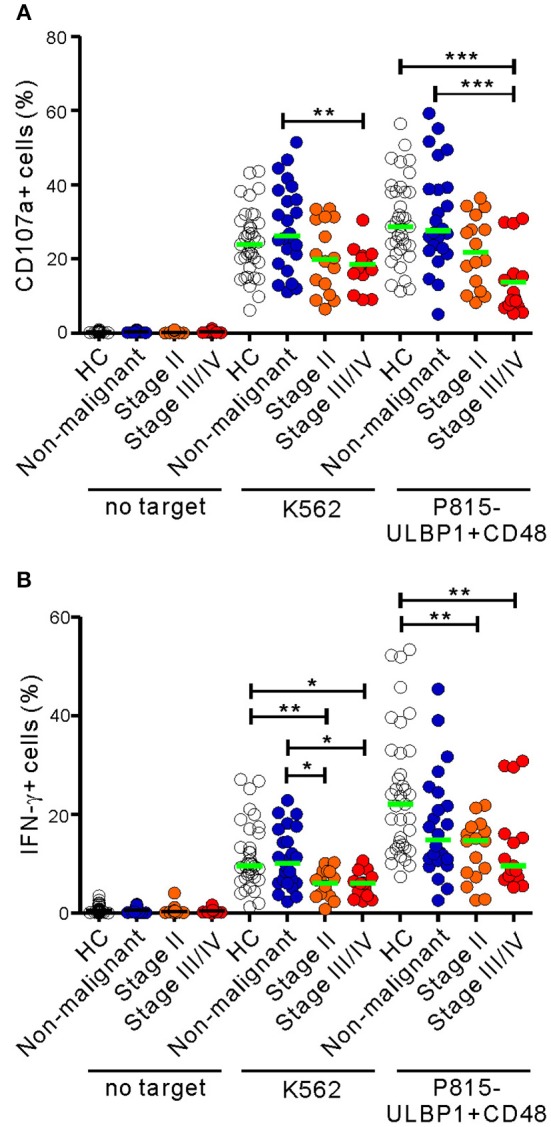
NK cell dysfunction correlates with the clinical severity of PC. The malignant group was divided into subgroups on the basis of cancer progression (stage II vs. stage III/IV). PBMCs from the healthy control (HC) group (*n* = 37), the non-malignant group (*n* = 24), the stage II subgroup (*n* = 16), and the stage III/IV subgroup (*n* = 15) were incubated with or without the indicated target cells, as described in Figure 1. The percentages of CD107a+ **(A)** and IFN-γ+ **(B)** NK cells are shown. Horizontal bars (green) denote the medians. *P < 0.05, **P < 0.01, and ***P < 0.001; Kruskal-Wallis test.

The 31 patients in the malignant group were classified on the basis of clinical stage into two subgroups, the early-stage group (stage II; *n* = 16) and the advanced-stage group (stage III/IV; *n* = 15) (Table S2). Compared with HCs, NK cells from the advanced-stage group exhibited a marked impairment of cytotoxic degranulation in response to P815-ULBP1+CD48 cells but not K562 cells (*P* < 0.001) (Figure 2A). This impairment was not seen in the early-stage group following receptor coactivation, suggesting the most severe defect in the advanced stage group. A significant impairment of NK cell degranulation was also observed in the advanced-stage group compared with the non-malignant group in response to both target cells (*P* < 0.01 against K562; *P* < 0.001 against P815-ULBP1+CD48). Similarly, as the stage of PC advanced, a significant and gradual decrease in NK cell IFN-γ expression following receptor coactivation was observed (Figure 2B). However, the frequencies of NK cell subsets with respect to CD56 and CD16 expression were comparable among the three groups (Figures S3, S4). These results suggest that a progressive impairment of NK cell function, but not NK cell frequency, is associated with advanced disease.

NK cell effector functions in the malignant group were next analyzed according to clinical parameters related to tumor progression (Tables S3, S4). Of interest, NK cell degranulation, but not IFN-γ expression, was significantly impaired in patients with poor prognostic factors, such as high serum levels of CA19-9 (>100 U/ml) (*P* < 0.05 against P815-ULBP1+CD48 by univariate analysis) and poorly differentiated high-grade tumors (*P* < 0.05 against both target cells by univariate analysis; *P* < 0.05 against P815-ULBP1+CD48 by multivariate analysis). By comparison, NK cell function did not differ according to tumor location or size, perineural invasion, lymphovascular invasion, or lymph node (LN) metastasis. These results suggest that impaired NK cell function, particularly cytotoxic degranulation, is associated with characteristics of tumor immune escape and progression.

### NK Cell Dysfunction Is Associated With Recurrence or Survival Outcome in PC

**Figure 3:**
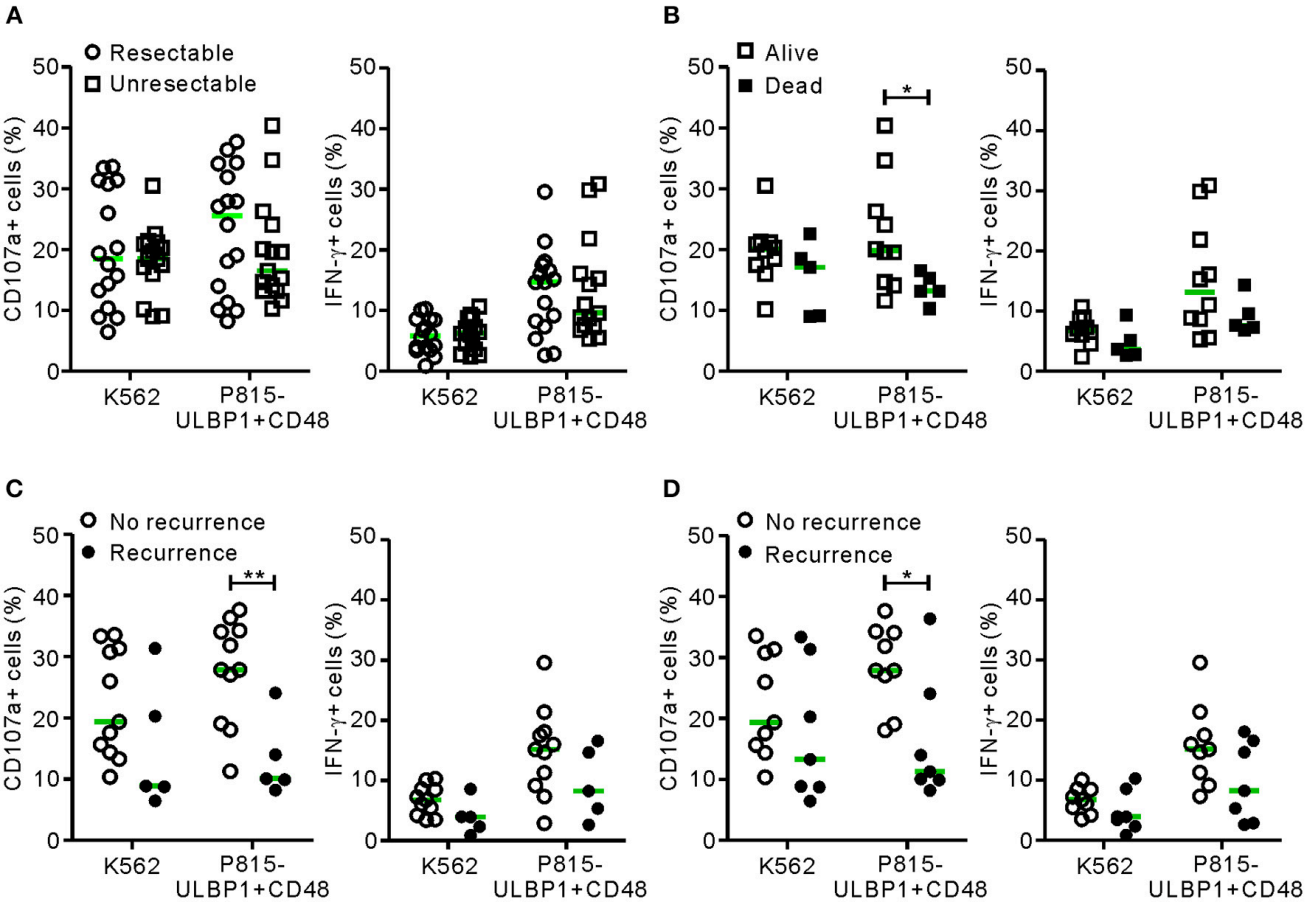
NK cell dysfunction correlates with clinical outcome in PC. **(A)** The malignant group was divided into resectable and unresectable subgroups. PBMCs from the resectable group (*n* = 16) and the unresectable group (*n* = 15) were incubated with or without the indicated target cells, as described in Figure 1 (circle, resectable; square, unresectable). In each group, NK cell surface expression of CD107a and intracellular expression of IFN-γ were compared. **(B)** The unresectable group was subdivided into living (*n* = 10) or deceased (*n* = 5) patients at 6 months after diagnosis. The NK cell activity of each subgroup was compared. **(C)** The resectable group was subdivided into patients with (*n* = 11) or without (*n* = 5) recurrence at 6 months after surgical resection. The NK cell activity of each subgroup was compared. **(D)** The resectable group was subdivided into patients with (*n* = 9) or without (*n* = 7) recurrence at 12 months after surgical resection. Horizontal bars (green) indicate the medians. *P < 0.05 and **P < 0.01; Mann-Whitney *U*-test.

The relationship between NK cell dysfunction and clinical outcome was next investigated in a prospective study of survival and recurrence. The same cohort of 31 patients (the malignant group) was divided into the resectable group (*n* = 16) and the unresectable group (*n* = 15) according to their suitability for surgical resection at diagnosis. NK cell functions in the unresectable group were slightly decreased but not significantly differ from those in the resectable group (Figure 3A). A survival analysis of the unresectable group for the 6 month follow-up period was performed. Patients with low NK cytotoxic degranulation, rather than low IFN-γ production, were associated with a poor survival outcome (*P* < 0.05 against P815-ULBP1+CD48) (Figure 3B). Similarly, in the resectable group, patients with low cytotoxic degranulation suffered from a high incidence of recurrence at 6 months (*P* < 0.01) (Figure 3C) and 1 year (*P* < 0.05) (Figure 3D) after surgery. Thus, impairment of NK cell cytotoxic degranulation at diagnosis was associated with poor survival and disease recurrence after surgical resection, suggesting the potential prognostic value of NK cytotoxic activity in PC.

### Altered Expression of NK Cell-Activating Receptors in Patients With PC

**Figure 4:**
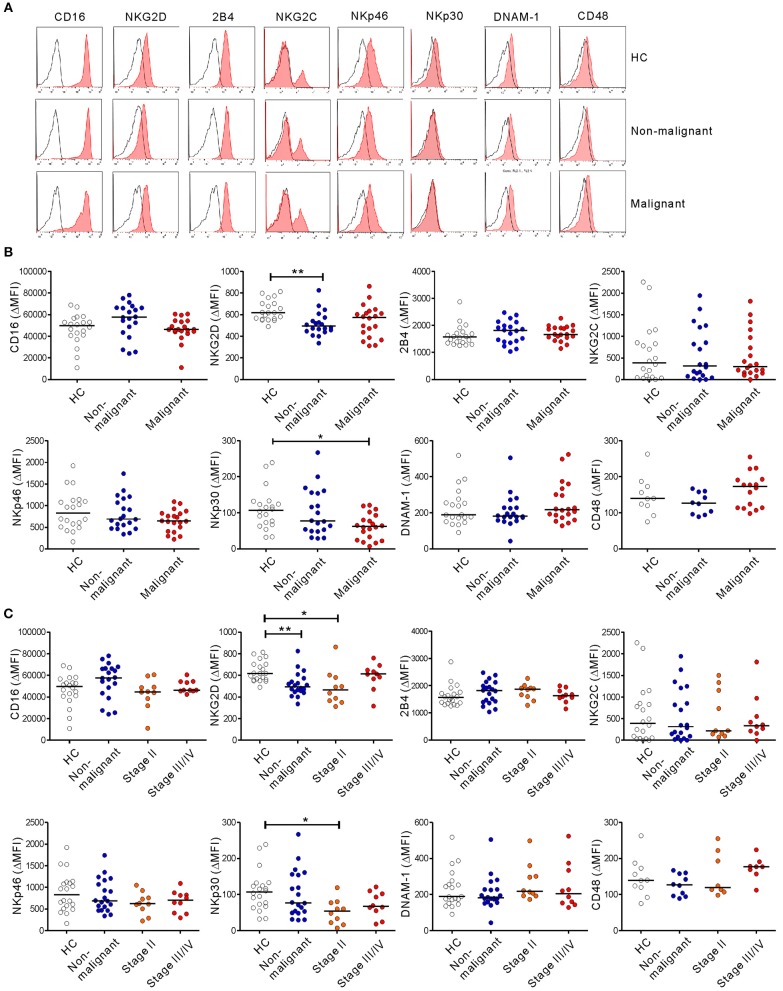
PC patients have altered expression of NK cell-activating receptors. **(A)** Representative FACS profiles showing the expression of CD16, NKG2D, 2B4, NKG2C, NKp46, NKp30, DNAM-1, and CD48 (red shaded histograms) on gated NK cells in the healthy control (HC) group, the non-malignant group, and the malignant group. The solid lines indicate isotype control staining. **(B)** The mean fluorescence intensity (MFI) of the expression of each indicated receptor on NK cells is shown relative to the MFI of the isotype control (ΔMFI) in the HC group (*n* = 20), the non-malignant group (*n* = 20), and the malignant group (*n* = 20). **(C)** The MFI of the expression of each indicated receptor on NK cells relative to the MFI of its isotype control (ΔMFI) in the HC group (*n* = 20), the non-malignant group (*n* = 20), the stage II subgroup (*n* = 10), and the stage III/IV subgroup (*n* = 10). Horizontal bars indicate the medians. *P < 0.05 and **P < 0.01; Kruskal-Wallis test.

NK cell activation against tumor cells is governed by the balance between activating and inhibitory receptors. A decrease or increase in these receptors often underlies NK cell dysfunction in various cancers, including PC (30). Thus, we assessed the expression of diverse activating receptors on NK cells. There were no significant differences in the expression of CD16, 2B4, NKG2C, NKp46, DNAM-1, or CD48 as a reference on NK cells among the three groups (Figure 4). The expression of NKp44 was nearly undetectable. Of note, compared with HCs, the expression of NKG2D on NK cells was significantly decreased in the non-malignant group (*P* < 0.01) and the early-stage group (*P* < 0.05), but not the advanced-stage group (Figure 4C). The level of NKp30 was also significantly decreased in the malignant group, specifically at the early stage, compared with HCs (*P* < 0.05). However, these significant differences (in ΔMFI) were not observed in terms of the percentage of positive cells (% values) (Figure S5). All the groups were comparable with regard to their expression of the inhibitory receptors KIR2DL1/S1, KIR2DL2/L3, and NKG2A (Figure S6).

**Figure 5:**
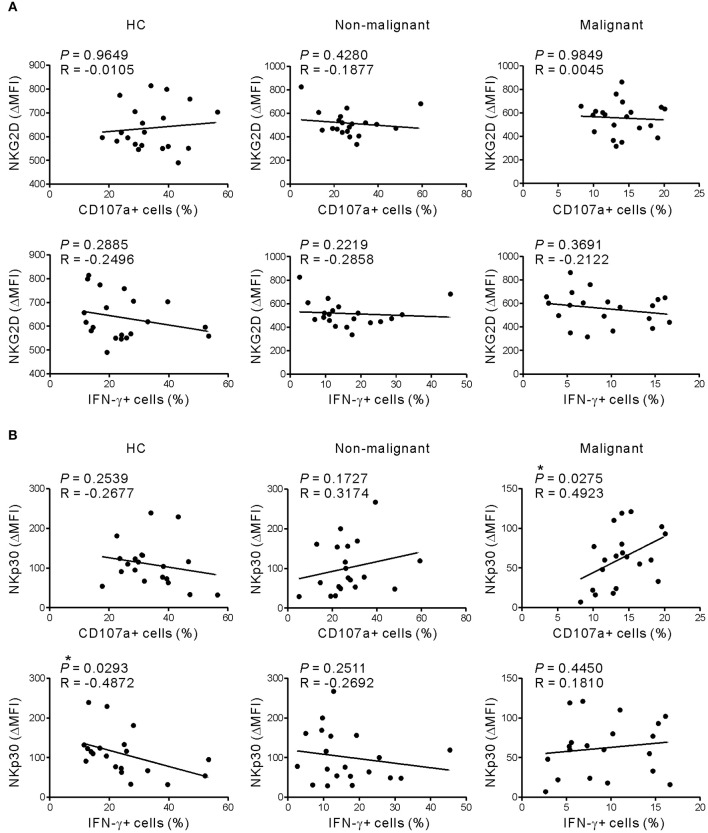
NKG2D expression does not correlate with NK cell dysfunction in response to receptor coactivation. **(A)** The expression of NKG2D (ΔMFI) was not correlated with the percentages of CD107a- or IFN-γ-positive NK cells after stimulation with P815-ULBP1+CD48 cells. **(B)** The expression of NKp30 (ΔMFI) correlated positively with the percentage of CD107a-positive NK cells in the malignant group after stimulation with P815-ULBP1+CD48 cells. *P < 0.05; Spearman's correlation test.

Given that NKG2D and NKp30 are implicated in PC (31, 32), the correlation between these receptors and NK cell function was assessed to evaluate their potential contribution to NK cell dysfunction. The expression of NKG2D did not correlate with the frequency of CD107a+ or IFN-γ+ NK cells following receptor coactivation in any group (Figure 5A and Figure S7A). By comparison, NKp30 expression was positively correlated with the frequency of CD107a+, but not IFN-γ+, NK cells in the malignant group (Figure 5B and Figure S7B). Of interest, the positive correlation between NKp30 expression and NK cell function was more evident after stimulation with K562 cells, which express the ligand for NKp30 receptor (26, 27). The lack of correlation between NKG2D level and NK cell function in the malignant group following activation with target cells engaging NKG2D however suggests that mechanisms other than NKG2D downregulation contribute to NK cell dysfunction in these patients.

### Altered Immunophenotype of NK Cells in Patients With PC

**Figure 6:**
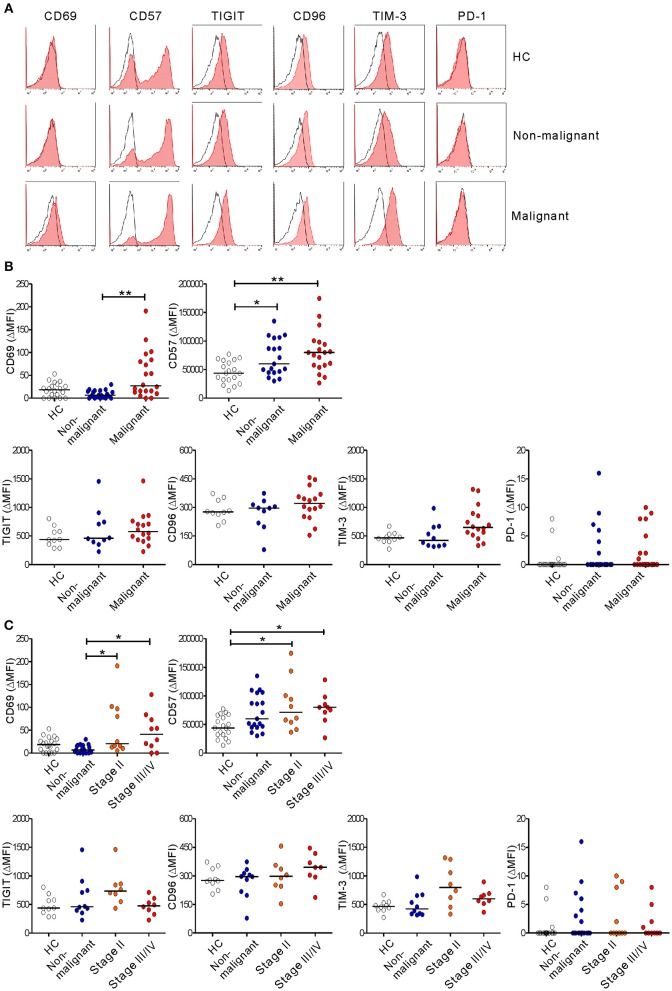
Altered immunophenotype of NK cells from PC patients. **(A)** Representative FACS profiles showing the expression of CD69, CD57, TIGIT, CD96, TIM-3, and PD-1 (red shaded histograms) on gated NK cells in the healthy control (HC) group, the non-malignant group, and the malignant group. The solid lines indicate the isotype control staining. **(B)** The MFI of the expression of the indicated receptors on NK cells relative to the MFI of the isotype control (ΔMFI) in the HC group (*n* = 20), the non-malignant group (*n* = 20), and the malignant group (*n* = 20). **(C)** The MFI of the expression of the indicated receptors on NK cells relative to the MFI of the isotype control on NK cells (ΔMFI) in the HC group (*n* = 20), the non-malignant group (*n* = 20), the stage II subgroup (*n* = 10), and the stage III/IV subgroup (*n* = 10). Horizontal bars indicate the medians. *P < 0.05 and **P < 0.01; Kruskal-Wallis test.

To further probe the phenotype of NK cells in PC, the expression of NK cell markers that reflect functional status was evaluated. The malignant group had significantly higher levels of the activation marker CD69 than did the non-malignant group, irrespective of disease stage (in ΔMFI) (Figure 6) and at the advanced stage (in % values) (Figure S8). In addition, the level of CD57, a marker for terminally differentiated cytotoxic NK cells (40), was significantly upregulated in the non-malignant group and the malignant group compared with HCs. Next, the expression of several immune checkpoint receptors that are associated with activation and/or exhaustion of lymphocytes in various cancers was assessed (35, 36). The expression of TIGIT and CD96 was comparable among the three groups, despite a slight upregulation of TIGIT in the early-stage group and of CD96 in the advanced-stage group. Of interest, there was a significant difference in the expression of TIM-3 on NK cells among the medians of the three groups (*P* < 0.05) without significant pairwise differences. However, the expression of PD-1 was hardly detectable on NK cells. Together with impaired effector function, the concomitant upregulation of markers related to activation, maturation, and/or exhaustion suggests phenotypic and functional alterations of NK cells during PC progression.

### TGF-β1 Levels Are Associated With Impaired NK Cell Degranulation in PC

**Figure 7:**
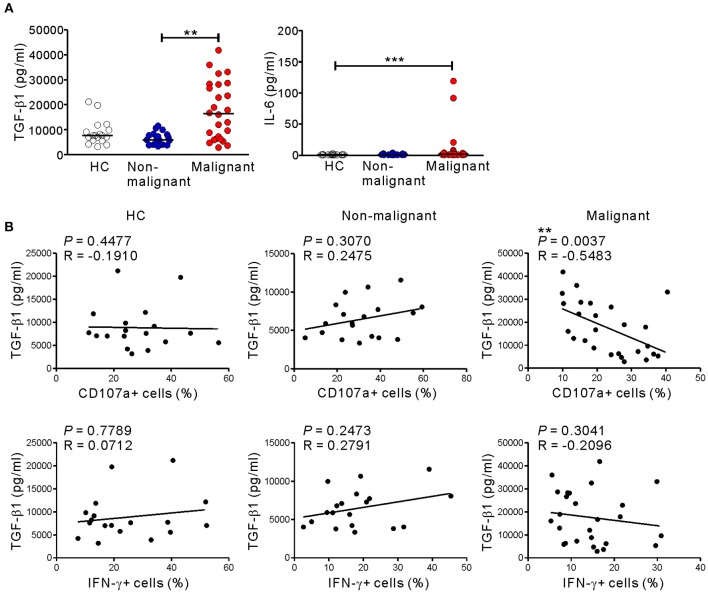
Correlation between the level of TGF-β1 and impaired NK cytotoxicity in PC patients. **(A)** The plasma levels of TGF-β1 and IL-6 were measured by Luminex multiplex assay. Plasma levels of the indicated cytokines in the healthy control (HC) group (*n* = 18), the non-malignant group (*n* = 19), and the malignant group (*n* = 26). Horizontal bars denote the medians. **(B)** Plasma TGF-β1 levels in the malignant group correlated inversely with the percentages of CD107a-positive NK cells after stimulation with P815-ULBP1+CD48 cells. **P < 0.01 and ***P < 0.001; Kruskal-Wallis with Dunn's test (A) and Spearman's correlation test (B).

Because the altered phenotype and compromised function of NK cells in the malignant group was unrelated to NKG2D receptor downregulation (Figure 4), we hypothesized the involvement of soluble mediator(s). Thus, we assessed the levels of various cytokines in the plasma of the study groups using a multiplex cytokine assay. The malignant group had significantly higher levels of IL-6 and, most notably, TGF-β1, a potent inhibitor of NK cell effector functions (41) (Figure 7A). By comparison, the levels of other cytokines including TNF-α, TGF-β2, TGF-β3, IL-4, IL-5, IL-10, and IL-13 were below the detection limit in all the groups (Table S5). Of note, the levels of TGF-β1 in the malignant group inversely correlated with the frequencies of CD107a+, but not IFN-γ+, NK cells after stimulation with P815-ULBP1+CD48 cells (*P* < 0.01) (Figure 7B) or K562 cells (*P* < 0.05) (Figure S9). In support of the elevated plasma levels of TGF-β1, a significant increase in the area and intensity of TGF-β1 staining around the pancreatic ducts was detected in malignant tissue compared with adjacent normal tissue within pancreatic tumor specimens (Figure S10).

## Discussion

In PC, less attention has been paid to NK cells than other immune effector cells (42), and their therapeutic potential has only recently been appreciated (30). A major difficulty in understanding the role of NK cells in PC is the conflicting data on the relationship between the frequency and/or activity of NK cells and prognosis (21–25). Considering the high variability in NK cell frequencies among human patients (14), the conflicting observations in previous studies might be due to the use of methods that measure NK cell activity within bulk PBMC preparations rather than the methods employed here, which measure NK cell activity on a per-cell basis. Here, we found a significant decrease in the function, but not the frequency, of circulating NK cells in PC and its progressive impairment during PC progression. Of note, a defect in NK cell cytotoxic degranulation, rather than IFN-γ production, was associated with poor prognostic factors such as poorly differentiated high-grade tumors, cancer recurrence, and mortality. These findings highlight the importance of a measurement of individual NK cell function and frequency in relation to clinical prognosis in PC.

Progressive impairment of NK cell function was more evident with the inclusion of a group of patients with non-malignant tumors, together with the stratification of the malignant group based on the disease stage. Compared with HCs, the non-malignant group exhibited decreased degranulation and a reduction in IFN-γ production (Figures 1B,D) following receptor coactivation, correlating with decreased NKG2D expression (Figure 4B). These findings suggest that NK cell dysfunction may already occur at the premalignant stage.

Of interest is the clinical finding that NK cell degranulation, but not IFN-γ production, was found to be predictive for patient prognosis in PC (Figure 3). A defect in cytotoxic degranulation at diagnosis was significantly linked to a high risk of cancer recurrence and mortality. In addition, impaired cytotoxic degranulation was associated with prognostic factors indicative of cancer progression (Tables S3, S4), compatible with the role of NK cells in the prevention of early tumorigenesis and progression (10, 11). By comparison, the contribution of IFN-γ production to PC prognosis was less clear, despite a slight impairment in patients with poor prognostic factors. IFN-γ has been shown to enhance cancer immunosurveillance in mouse models (43), but appears to play more complicated roles in humans (22, 44). Whereas an IFN-γ gene signature in tumors is related to a better prognosis (e.g., later recurrence) for resectable PC (44), a negative correlation between the frequency of IFN-γ+ NK cells and PC mortality was observed after pemetrexed therapy (22). These findings together suggest that NK cell cytotoxic degranulation, rather than IFN-γ production, correlate well with PC progression. This notion is also supported by a previous prospective study reporting a high incidence of various cancers in subjects with low NK cell cytotoxicity (15). However, given a relatively small sample size in this study, further study is required to validate our results with the inclusion of sufficient number of cases.

To our knowledge, this study is the first to demonstrate functional impairment of NK cells and its relation to cancer prognosis by employing target cells that stimulate NK cells via specific receptor-ligand interactions. Conventional K562 cells were also used as target cells to validate the functional impairment of PC NK cells. K562 cells are classically used to assess NK cell cytotoxic activity (15). They express ligands for NKG2D, DNAM-1, and NKp30 (26, 27). Given such multiple receptor-ligand interactions, functional deficiencies of NK cells might not be apparent when K562 cells are solely used as target cells. Indeed, cytotoxic degranulation of NK cells from X-linked lymphoproliferative disease (XLP1) patients was comparable to that of HC NK cells after stimulation with K562 cells, but was defective in response to Epstein-Barr virus (EBV)-transformed B-cell line 721.221 (37). Compared with K562 cells, stimulation of NK cells with P815-ULBP1+CD48 cells resulted in greater functional differences among study groups and demonstrated a greater prognostic value. Furthermore, it could enable us to assess whether PC NK cell dysfunction is associated with altered NKG2D expression. Thus, the assessment of NK cell function via stimulation with defined receptor-ligand interactions could confirm and/or complement results obtained with K562 cells.

It has been shown that downregulation of activating receptors is a major mechanism of NK cell dysfunction in various cancers, including PC (30). We observed significantly decreased expression of NKp30 and NKG2D on PC NK cells from the early-stage but not the advanced-stage group. The importance of these receptors in PC is supported by their association with clinicopathological features such as LN metastasis (32) and the preferential expression of their cognate ligands on PC cells (31, 45). There was a discernible trend toward decreased expression of NKp30, but not NKG2D, during PC progression (Figure 4B), correlating with reduced cytotoxic activity in the malignant group (Figure 5B and Figure S7B). These results suggest the involvement of NKp30 in PC progression, which merits further investigation. Of interest, the severe impairment of NK cell function in the advanced-stage group was unrelated to NKG2D downregulation (Figures 4C, 5A and Figure S7A), suggesting the potential involvement of other mediator(s) in NK cell dysfunction. Supporting this notion, we found a significant upregulation of CD69 and CD57, markers linked to activation and maturation, during PC progression despite the loss of NK cell function. Furthermore, we observed a similar upregulation of TIM-3, checkpoint receptors related to activation/exhaustion of lymphocytes (35, 36, 46). Although further study is required, these data suggest that NK cells upregulate activation and maturation markers but are rendered more responsive to immune checkpoint pathways during PC progression, corroborating a recent study of the characterization of PD-1+ NK cells co-expressing CD69 in mouse tumor models (47). TIM-3 is an activation-induced checkpoint receptor that is expressed on all mature NK cells and further upregulated upon activation. TIM-3 is also upregulated on circulating NK cells from patients with various cancers (46, 48) and associated with NK cell exhaustion in advanced melanoma (46) and lung adenocarcinoma (48). Collectively, these findings suggest that NK cells may undergo concomitant phenotypic and functional alterations leading to the loss of effector function during PC progression.

Cancer cells, including PC cells, can also impair NK cell function via the release of soluble factors (9, 42, 49). Consistent with previous studies (9, 50), significantly elevated levels of TGF-β1 were detected in the plasma and tumor tissue of PC patients. Furthermore, the inverse correlation between TGF-β1 levels and NK cell degranulation suggests that TGF-β1 deregulation is linked to the impaired cytotoxic activity. Tumor-associated TGF-β1 has been shown to play a crucial role in the downregulation of anti-tumor immune responses mediated by diverse immune cells, including NK cells (9, 49). TGF-β1 also induces proliferation and survival of PDAC cells in the advanced stage and promotes epithelial-to-mesenchymal transition (EMT), invasion, and metastasis (49). In this respect, TGF-β1 may generate a favorable environment for tumor growth through a suppression of anti-tumor immunity including NK cell function, beyond its direct tumor-promoting effects. Given the association of TGF-β1 with poor survival in advanced PDAC (50) and poor response to checkpoint blockade in metastatic cancers (51), TGF-β1 may be an appropriate target for therapeutic intervention in PC, particularly at the advanced stage.

In summary, the present study showed a progressive impairment of NK cell functions during PC progression. This NK cell dysfunction, particularly impaired cytotoxic degranulation, was associated with known prognostic factors indicative of disease progression and correlated with cancer recurrence and mortality. Due to the challenges in accessing the tumor tissue in advanced-stage patients, assessment of circulating NK cells may have a prognostic value. Although further validation is required, the present study suggests that NK cell function, especially cytotoxic degranulation, is a complementary and previously unappreciated prognostic factor in PC. Our findings may also provide support for therapies aimed at restoring or enhancing immune surveillance by NK cells in PC.

## Data Availability

The datasets for this manuscript are not publicly available because of sensitive information. The raw data supporting the conclusions of this manuscript will be made available by the authors to qualified researchers upon reasonable request. Requests to access the datasets should be directed to hunkim@amc.seoul.kr.

## Ethics Statement

The study complied with the Declaration of Helsinki, and the protocol was reviewed and approved by the Institutional Review Board (IRB) of Asan Medical Center (IRB No. 2016-0865). The study was also registered as a clinical study (NCT03665571) with the Clinical Research Information Service (available at https://clinicaltrials.gov). Written informed consent was acquired from all participants.

## Author Contributions

EJ, AS, J-WC, HL, M-YK, and HJK were involved in data acquisition. EJ, AS, HJK, and YB were involved in data analysis and interpretation. D-HK and SCK provided blood samples with informed consent and helped design studies with blood samples. SWK, YB, SCK, and HSK contributed to the conceptual design of the study and writing of the manuscript with an input from all co-authors.

### Conflict of Interest Statement

HSK and SCK are inventors on patent application (1020170033207) in Korea submitted by University of Ulsan College of Medicine that covers the use of receptor coactivation for diagnosis. The authors have not conducted any paid or unpaid consultation regarding this work. The remaining authors declare that the research was conducted in the absence of any commercial or financial relationships that could be construed as a potential conflict of interest.
