# Supplementary material for: Progressive Impairment of NK Cell Cytotoxic Degranulation Is Associated With TGF-β1 Deregulation and Disease Progression in Pancreatic Cancer
Source: Front Immunol. 2019 Jun 21;10:1354. doi: 10.3389/fimmu.2019.01354 (PMC6598013; doi:10.3389/fimmu.2019.01354)
Supplement: Supplementary file 1 [file Data_Sheet_1.docx]

Supplementary Material

**Progressive impairment of NK cell cytotoxic degranulation is associated with TGF-β1 deregulation and disease progression in pancreatic cancer**

Eunsung Jun^*^, Ah Young Song^*^, Ji-Wan Choi, Hyeon Ho Lee, Mi-Yeon Kim, Dae-Hyun Ko, Hyo Jeong Kang, Seong Who Kim, Yenan Bryceson, Song Cheol Kim^#^, and Hun Sik Kim^#^

**Running Title:** NK cell dysfunction as a prognostic factor in pancreatic cancer

*These authors contributed equally to this work

**#**Correspondence: Song Cheol Kim, E-mail: drksc@amc.seoul.kr; and Hun Sik Kim, E-mail: hunkim@amc.seoul.kr

**
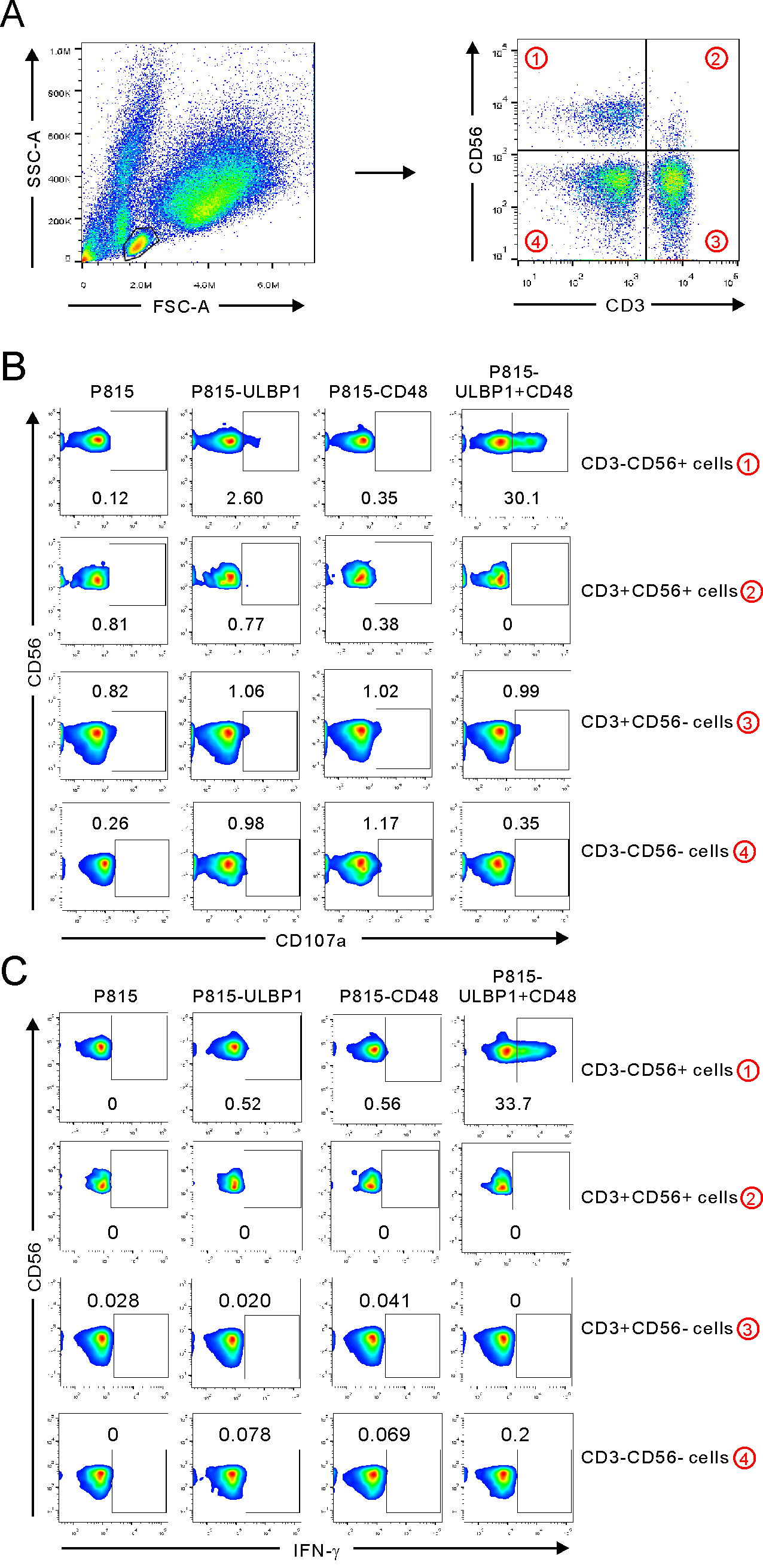
**

**Figure S1. Selective stimulation of NK cells via NKG2D and 2B4.**

(A) Representative FACS profiles showing the strategy for identifying CD3-CD56+ NK cells within the lymphocyte gate. (B, C) PBMCs were mixed with P815 cells expressing ULBP1, a ligand for NKG2D, and/or CD48, a ligand for 2B4 (P815 control, P815-ULBP1, P815-CD48, or P815-ULBP1+CD48 cells). Representative FACS profiles showing the percentages of CD107a-positive cells after 2 hours of stimulation (B) and the percentages of IFN-γ-positive cells after 6 hours of stimulation (C) in the indicated cell populations.


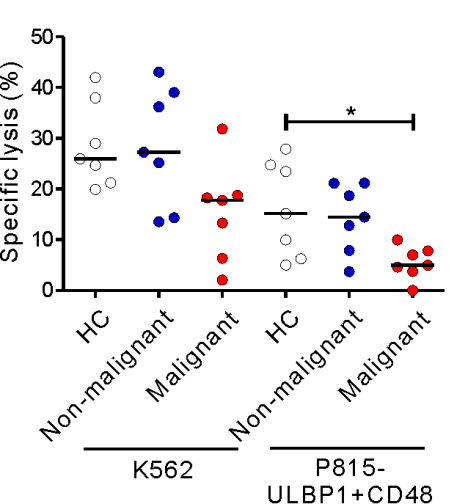


**Figure S2. Patients with pancreatic cancer (PC) have impaired NK cell cytotoxicity.**

Comparison of the lysis of K562 and P815-ULBP1+CD48 cells by resting NK cells in PBMCs from the HC group, the non-malignant group, and the malignant group. Results were obtained from seven different donors in each group at the effector-to-target (E:T) ratio of 50:1. Horizontal bars indicate the medians. **P* < 0.05; Kruskal-Wallis test.


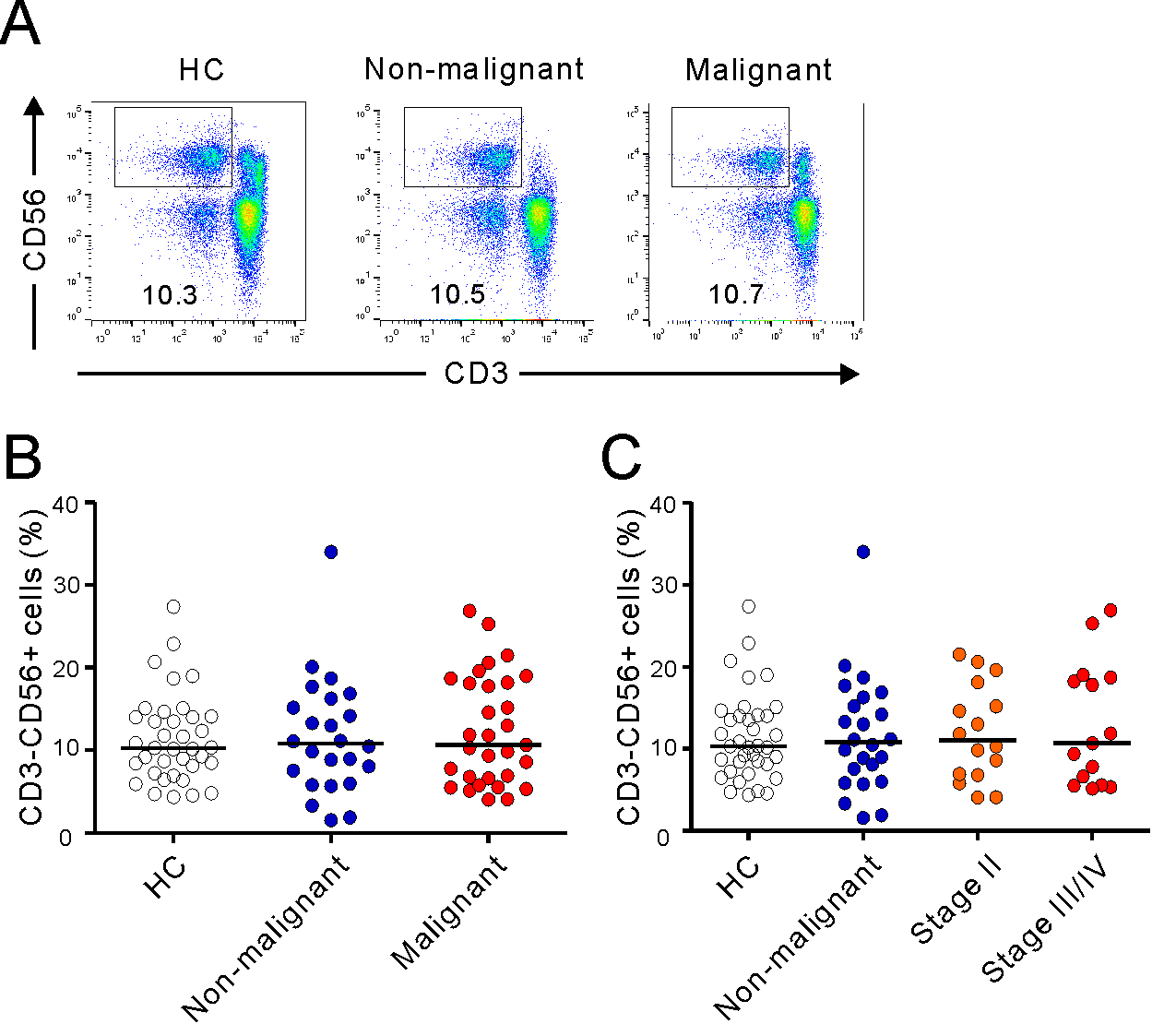


**Figure S3. PC patients have comparable NK cell frequencies to healthy controls.**

(A) Representative FACS profiles showing the frequency of CD3-CD56+ NK cells. (B) The percentages of total NK cells in the PBMCs from the healthy control (HC) group (n = 37), the non-malignant group (n = 24), and the malignant group (n = 31). (C) Comparison of the percentages of peripheral blood NK cells in the HC group, the non-malignant group, the stage II malignant subgroup (n = 16), and the stage III/IV malignant subgroup (n = 15).


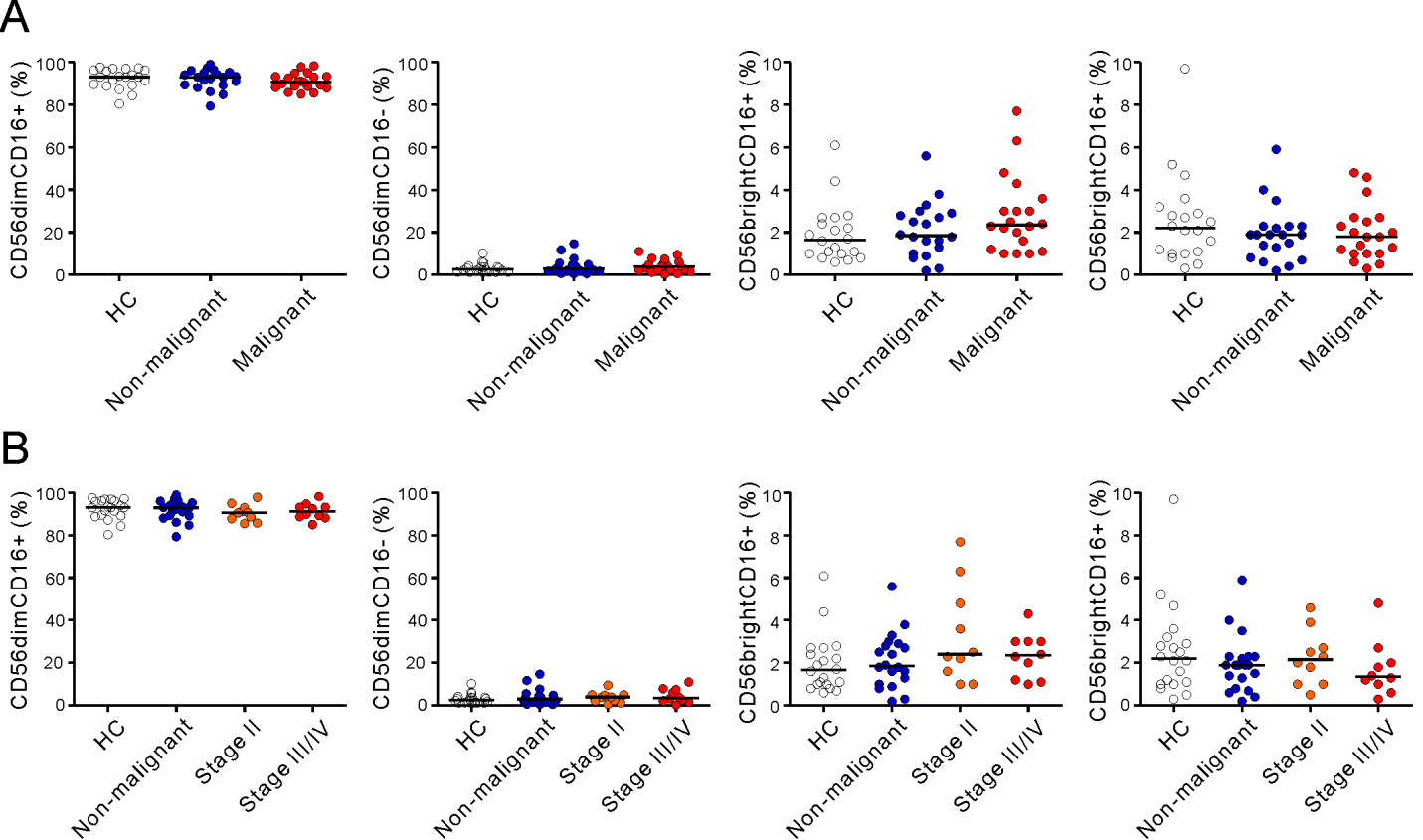


**Figure S4. The distribution of individual NK cell subsets in PC patients is comparable to that in healthy controls.**

(A, B) The distribution of NK cell subsets (CD56^dim^CD16+, CD56^dim^CD16-, CD56^bright^CD16+, and CD56^bright^CD16-) in the healthy control (HC) group (n = 20), the non-malignant group (n = 20), and the malignant group (n = 20) (A) or in the stage II (n = 10) and stage III/IV malignant subgroups (n = 10) (B).


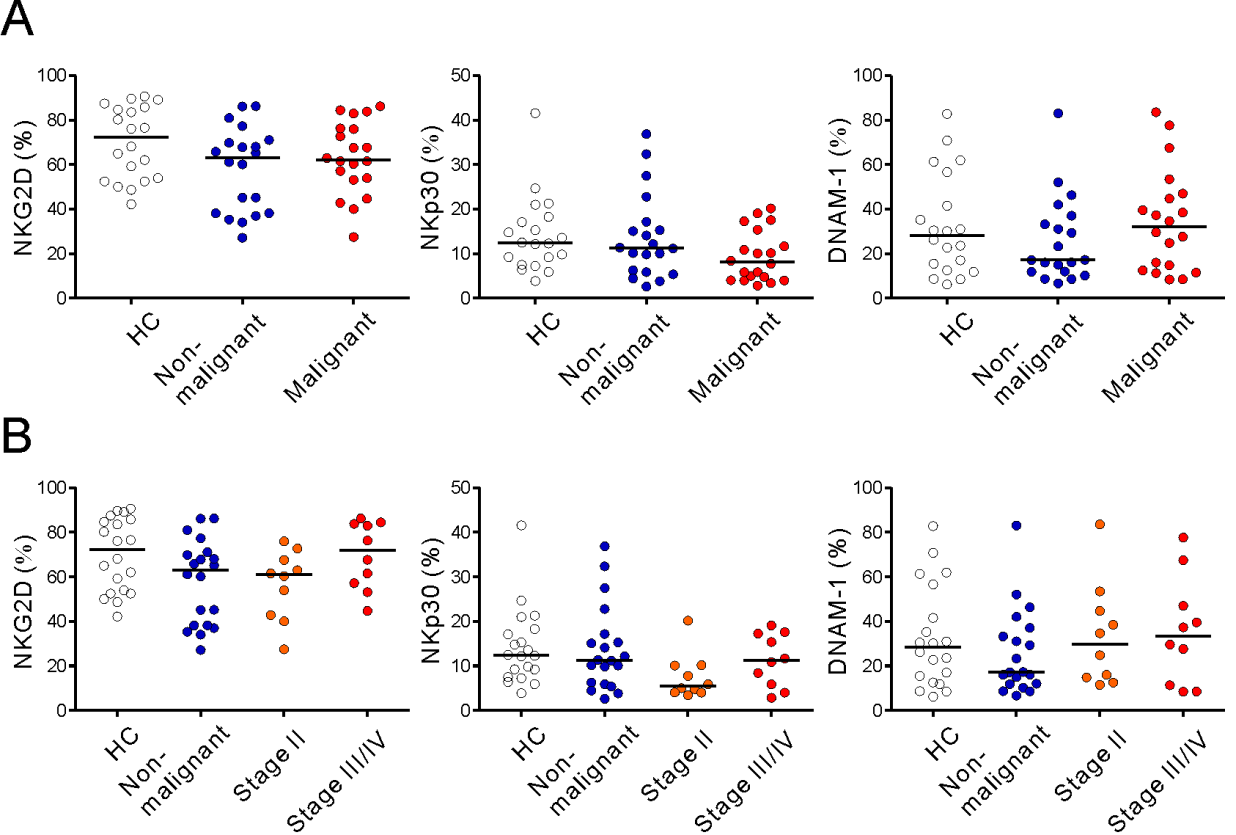


**Figure S5. Comparison of NKG2D, NKp30 or DNAM-1-positive NK cells among study groups.**

Comparison of the percentages of NK cells for the expression of the indicated receptors in the HC group (n = 20), the non-malignant group (n = 20), and the malignant group (n = 20) (A), or the stage II malignant (n = 10) and stage III/IV malignant subgroups (n = 10) (B). Horizontal bars indicate the medians.


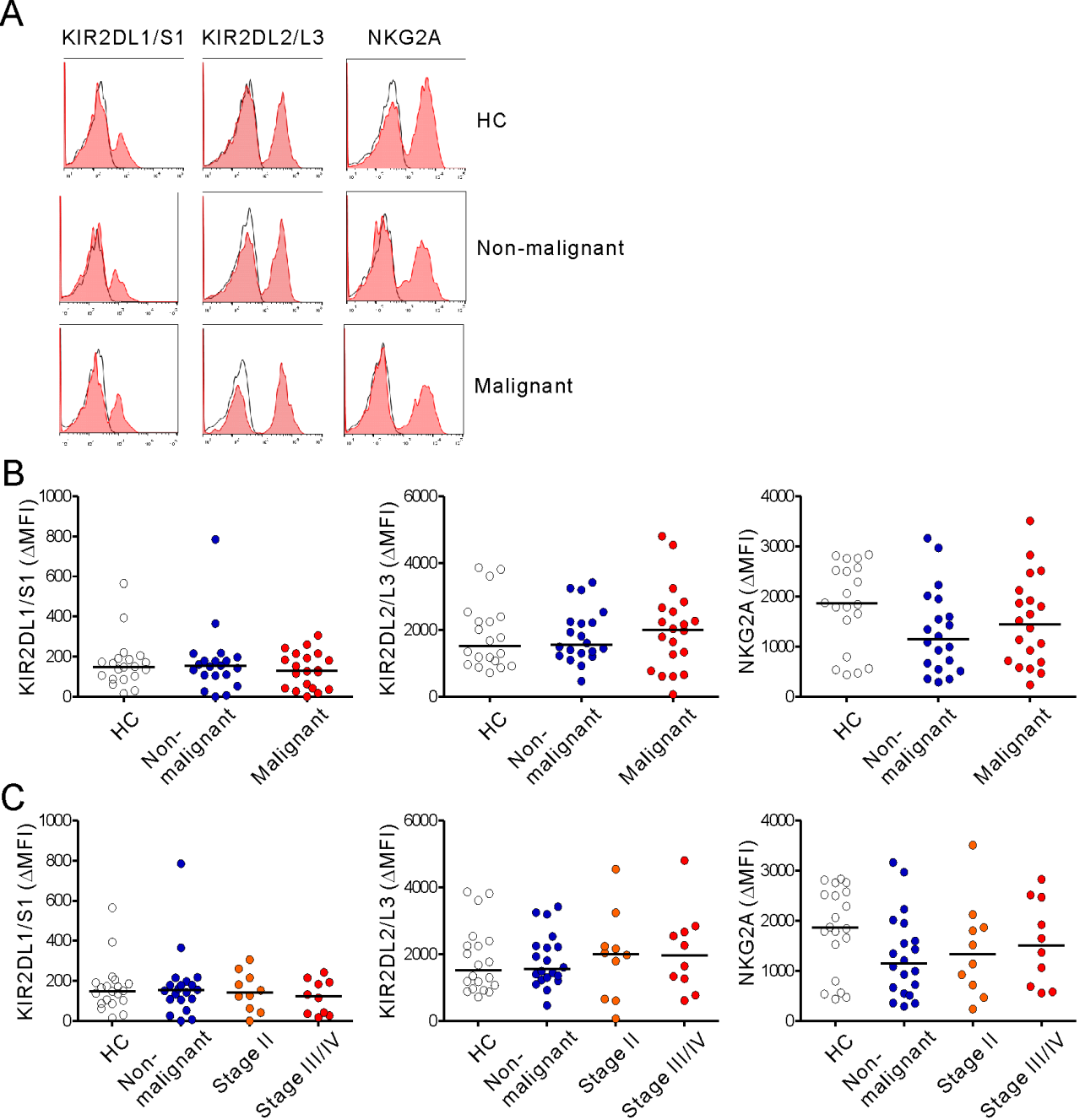


**Figure S6. The expression of NK cell inhibitory receptors in PC patients is comparable to that in healthy controls.**

(A) Representative FACS profiles showing the expression of KIR2DL1/S1, KIR2DL2/L3, and NKG2A (red shaded histograms) on gated NK cells in the healthy control (HC) group, the non-malignant group, and the malignant group. The solid lines indicate the staining of the isotype control. (B, C) Comparison of the ΔMFI for the expression of the indicated receptors in the HC group (n = 20), the non-malignant group (n = 20), and the malignant group (n = 20) (B), or the stage II malignant (n = 10) and stage III/IV malignant subgroups (n = 10) (C). Horizontal bars indicate the medians.


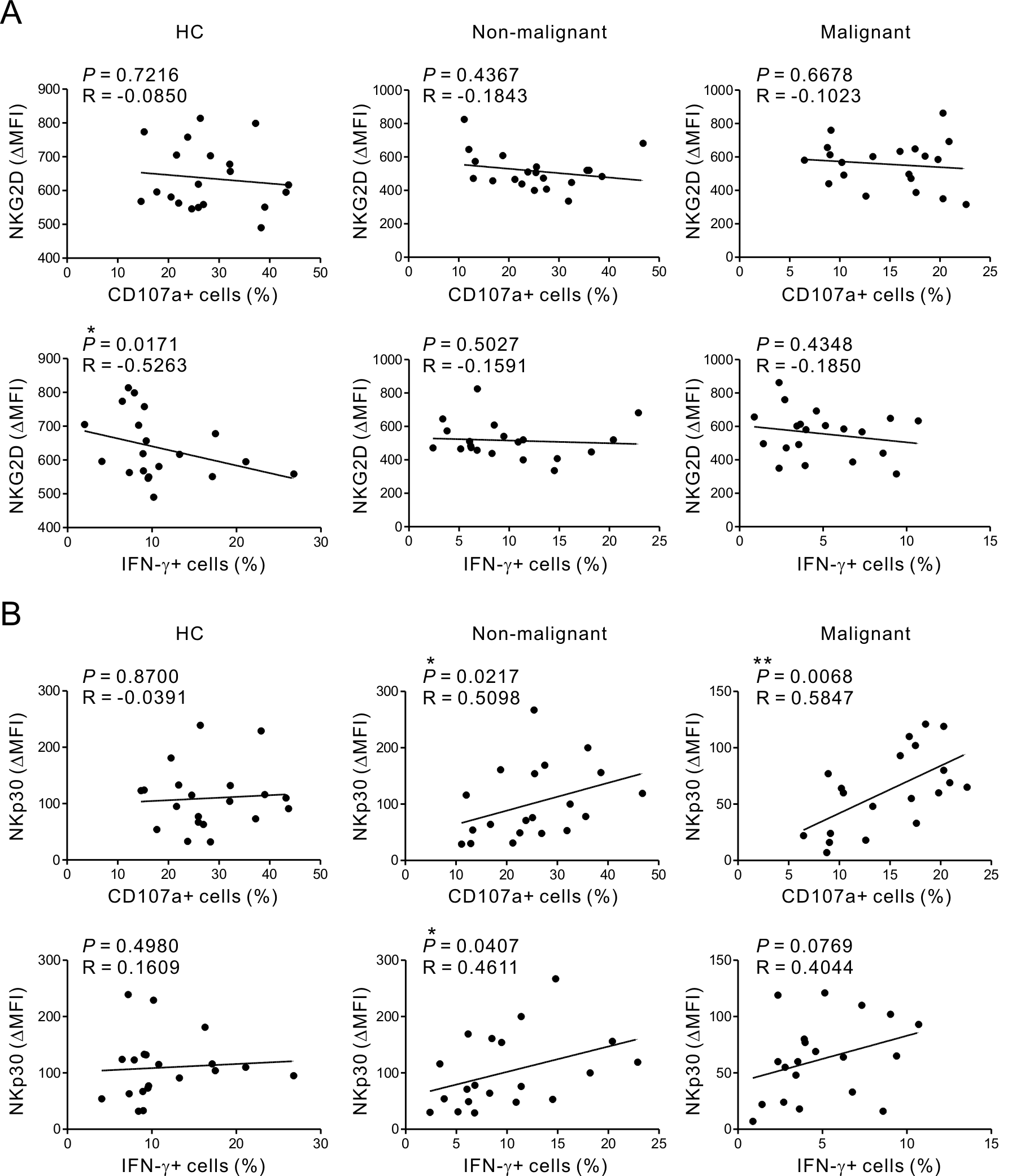


**Figure S7. NKp30 but not NKG2D expression correlates with impaired NK cell functions.**

(A) The expression of NKG2D (ΔMFI) did not correlate with the percentages of CD107a- or IFN-γ-positive NK cells after stimulation with K562 target cells. (B) The expression of NKp30 (ΔMFI) correlated positively with the percentage of CD107a-positive NK cells in the non-malignant and malignant groups after stimulation with K562 cells. **P* < 0.05 and ***P* < 0.01; Spearman’s correlation test.


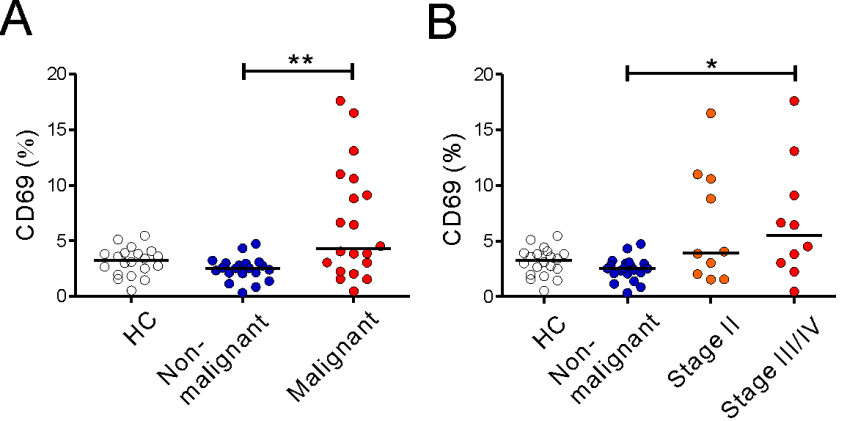


**Figure S8. CD69-positive NK cells are increased in PC patients.**

Comparison of the percentages of CD69-positive NK cells in the HC group (n = 20), the non-malignant group (n = 20), and the malignant group (n = 20) (A), or the stage II malignant (n = 10) and stage III/IV malignant subgroups (n = 10) (B). Horizontal bars indicate the medians. **P* < 0.05 and ***P* < 0.01; Kruskal-Wallis test.


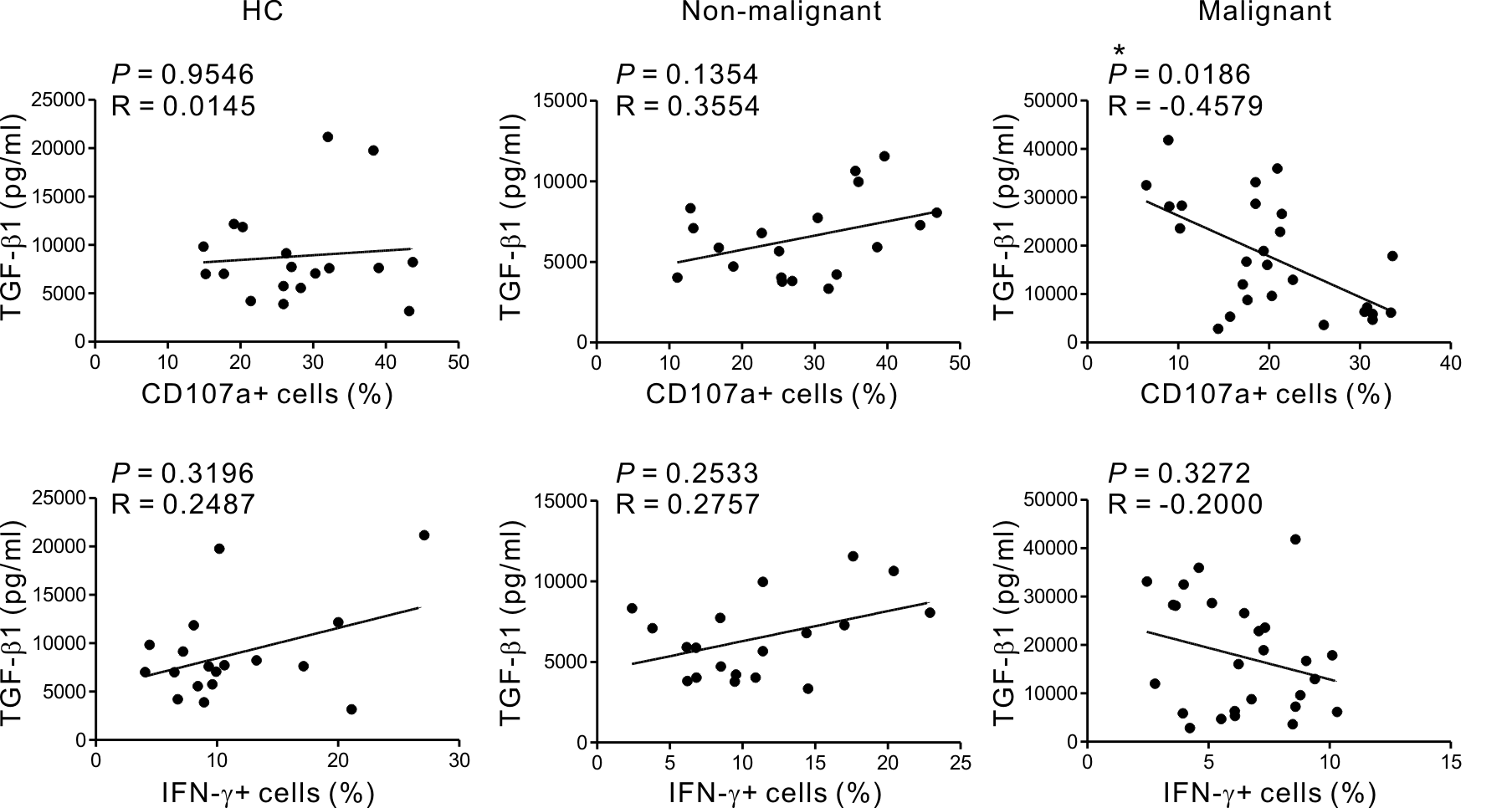


**Figure S9. Association between TGF-β1 levels and impaired NK cell cytotoxicity.**

TGF-β1 levels in the plasma of patients in the malignant group correlated inversely with the percentages of CD107a-positive NK cells after stimulation with K562 target cells. **P* < 0.05; Spearman’s correlation test.


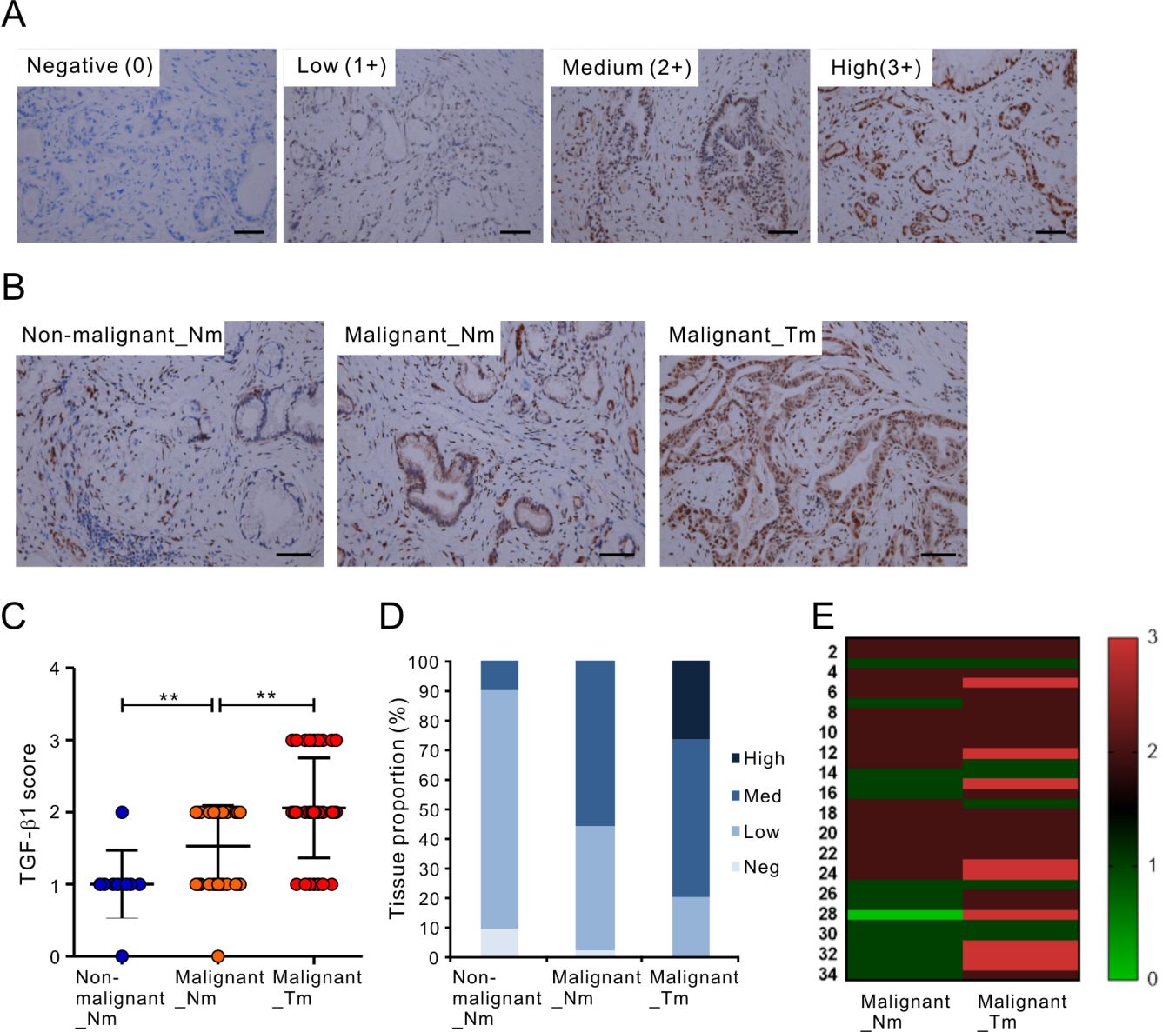


**Figure S10. Patients with PC have increased levels of tumor-associated TGF-β1 expression.**

(A) Human pancreatic cancer tissue was stained for TGF-β1. The intensity of the staining was scored independently by two pathologists (score: 0–3). (B) Representative image of TGF-β1 staining in non-malignant and malignant tumors (Nm, normal tissue; Tm, tumor tissue). (C) Comparison of scoring for TGF-β1 expression among the three groups. (D) Comparison of the staining pattern for TGF-β1 expression among the three groups. (E) TGF-β1 expression in paired normal and tumor tissue from individual patients is shown as a heat map. ***P* < 0.01; Mann-Whitney *U*-test.
