# Supplementary material for: Progressive Impairment of NK Cell Cytotoxic Degranulation Is Associated With TGF-β1 Deregulation and Disease Progression in Pancreatic Cancer
Source: Front Immunol. 2019 Jun 21;10:1354. doi: 10.3389/fimmu.2019.01354 (PMC6598013; doi:10.3389/fimmu.2019.01354)
Supplement: Supplementary file 2 [file Data_Sheet_2.docx]

**Table S1. Demographic data of the healthy controls (HCs), patients with non-malignant lesions (non-malignant group), and patients with pancreatic cancer (malignant group).**

|  | HC | Non-malignant group | Malignant group | *P*-value |
| --- | --- | --- | --- | --- |
| Number of subjects | 37 | 24 | 31 |  |
| Median age (Range) | 28.0 (20.0 – 38.0) | 54.7 (28.0 – 71.0) | 69.0 (36.0 – 81.0) | <0.001 |
| Gender (Female/Male) | 25/12 | 20/4 | 16/15 | 0.046 |
| NK cell (%)* | 10.3 (4.4 – 27.4) | 10.8 (1.57 – 34.0) | 10.7 (4.08 – 26.9) | 0.748 |

*Values are presented as the median (range).

**Table S2. Clinicopathological characteristics of patients in the non-malignant and malignant groups.**

|  | Non-malignant group | Malignant group | Univariate  *P*-value | Multivariate  *P*-value |
| --- | --- | --- | --- | --- |
| Number of subjects | 24 | 31 |  |  |
| Median age (Range) | 54.7 (28.0–71.0) | 69.0 (36.0–81.0) | 0.003 |  |
| Gender (Female/Male) | 20/4 | 16/15 | 0.014 |  |
| BMI (kg/m^2^)* | 24.2 (18.9–29.7) | 23.1 (15.9–45.9) | 0.646 |  |
| Hb (g/dl)* | 12.9 (9.6–16.4) | 12.6 (7.9–15.2) | 0.238 |  |
| WBC (10^3/μL)* | 6.3 (4.1–10.1) | 5.9 (3.2–12.2) | 0.931 |  |
| Neutrophil (%)* | 56. (45.2–71.6) | 61.0 (42.4–81.1) | 0.063 |  |
| Lymphocyte (%)* | 33.3 (22.3–46.3) | 26.9 (8.7–42.8) | 0.005 | 0.040 |
| Monocyte (%)* | 6.4 (4.5–11.2) | 8.5 (4.4–14.6) | 0.007 |  |
| NK cell (%)* | 10.8 (1.57–34.0) | 10.7 (4.08–26.9) | 0.531 |  |
| CA19-9 (U/mL)* | 8.0 (0.6–55.9) | 143.6 (2.7–10972.0) | 0.052 | 0.013 |
| Diagnosis |  |  |  |  |
| Pancreas ductal adenocarcinoma |  | 30 (96.8%) |  |  |
| Mucinous adenocarcinoma |  | 1 (3.2%) |  |  |
| Intraductal papillary mucinous neoplasm (IPMN) | 10 (41.7%) |  |  |  |
| Mucinous cystic neoplasm (MCN) | 5 (20.8%) |  |  |  |
| Serous cystadenoma (SCA) | 4 (16.7%) |  |  |  |
| Neuroendocrine tumors (NET) | 3 (12.5%) |  |  |  |
| Acinar cell cystadenoma (ACA) | 1 (4.2%) |  |  |  |
| Solid pseudopapillary neoplasm (SPN) | 1 (4.2%) |  |  |  |
| Surgical resection |  |  |  |  |
| Total pancreatectomy | 1 (4.2%) | 0 (0.0%) |  |  |
| Pancreatoduodenectomy (PD) | 8 (33.3%) | 7 (22.6%) |  |  |
| Distal pancreatectomy and splenectomy (DPS) | 15 (62.5%) | 9 (29.0%) |  |  |
| None (unresectable) | 0 (0.0%) | 15 (48.4%) |  |  |
| Cancer stage |  |  |  |  |
| Early stage, II | Not valid | 16 (51.6%) |  |  |
| Advanced stage, III/IV | Not valid | 15 (48.4%) |  |  |

*Values are presented as the median (range).

**Table S3. Comparison of NK cell activity with clinical parameters in the malignant group (n = 31).**

| Factors | No. of patient | K562 stimulation | | | | | | P815-ULBP1+CD48 stimulation | | | | | |
| --- | --- | --- | --- | --- | --- | --- | --- | --- | --- | --- | --- | --- | --- |
|  |  | CD107a+ (%) | | | IFN-γ+ (%) | | | CD107a+ (%) | | | IFN-γ+ (%) | | |
|  |  | Mean ± SD | *P*-value | | Mean ± SD | *P*-value | | Mean ± SD | *P*-value | | Mean ± SD | *P*-value | |
|  |  |  | Univariate | Multivariate |  | Univariate | Multivariate |  | Univariate | Multivariate |  | Univariate | Multivariate |
| Age (yr) | | | | | | | | | | | | | |
| >60 | 11 | 18.2±6.4 | 0.775 | 0.331 | 5.1±2.8 | 0.745 | 0.828 | 19.1±8.7 | 0.941 | 0.889 | 11.3±6.9 | 0.732 | 0.560 |
| <60 | 20 | 19.7±8.8 |  |  | 6.3±2.7 |  |  | 19.6±10.2 |  |  | 12.9±8.1 |  |  |
| Gender | | | | | | | | | | | | | |
| Female | 16 | 19.2±7.2 | 0.968 | 0.450 | 5.8±2.6 | 0.590 | 0.829 | 19.9±9.1 | 0.716 | 0.125 | 13.3±7.9 | 0.628 | 0.244 |
| Male | 15 | 18.5±8.8 |  |  | 6.8±2.9 |  |  | 16.5±10.3 |  |  | 11.0±7.5 |  |  |
| BMI (kg/m^2^) | | | | | | | | | | | | | |
| <23 | 15 | 17.1±4.2 | 0.047 | 0.019 | 6.1±2.9 | 0.294 | 0.356 | 18.1±10.2 | 0.804 | 0.877 | 11.0±9.7 | 0.876 | 0.541 |
| >23 | 16 | 21.3±9.6 |  |  | 6.3±2.4 |  |  | 21.9±9.2 |  |  | 13.0±5.3 |  |  |
| Hb (g/dl) | | | | | | | | | | | | | |
| >13 | 13 | 20.3±8.6 | 0.096 | 0.184 | 6.8±3.1 | 0.586 | 0.995 | 27.9±10.4 | 0.026 | 0.094 | 14.7±8.7 | 0.335 | 0.311 |
| <13 | 18 | 17.3±6.9 |  |  | 5.8±2.4 |  |  | 15.9±7.7 |  |  | 11.2±6.7 |  |  |
| WBC (10^3^/µl) | | | | | | | | | | | | | |
| >4, <10 | 29 | 18.8±7.9 | 0.392 | 0.648 | 6.1±2.7 | 0.410 | 0.553 | 19.8±9.6 | 0.169 | 0.221 | 14.3±7.6 | 0.197 | 0.197 |
| <4 or 10< | 2 | 14.5±7.6 |  |  | 4.5±2.5 |  |  | 12.4±1.1 |  |  | 6.5±1.6 |  |  |
| Neutrophil (%) | | | | | | | | | | | | | |
| >50, <75 | 28 | 18.5±8.1 | 0.706 | 0.742 | 6.2±2.8 | 0.531 | 0.543 | 19.9±10.0 | 0.432 | 0.339 | 12.8±7.6 | 0.813 | 0.522 |
| <50 or 75< | 3 | 20.9±6.2 |  |  | 4.6±1.8 |  |  | 18.1±2.8 |  |  | 9.2±8.6 |  |  |
| Lymphocyte (%) | | | | | | | | | | | | | |
| >20, <44 | 26 | 18.1±8.5 | 0.958 | 0.376 | 6.2±2.6 | 0.864 | 0.933 | 19.9±9.6 | 0.344 | 0.660 | 14.4±6.8 | 0.418 | 0.434 |
| <20 or 44< | 5 | 19.7±3.5 |  |  | 5.1±3.2 |  |  | 14.0±9.6 |  |  | 6.8±11.4 |  |  |
| CA19-9 (U/mL) | | | | | | | | | | | | | |
| < 37 | 7 | 19.4±8.8 | 0.304 | 0.848 | 7.3±3.9 | 0.767 | 0.798 | 27.9±11.0 | 0.064 | 0.777 | 15.3±9.0 | 0.719 | 0.420 |
| > 37 | 24 | 18.1±7.6 |  |  | 6.1±2.3 |  |  | 17.3±8.6 |  |  | 11.2±7.3 |  |  |
| CA19-9 (U/mL) | | | | | | | | | | | | | |
| <100 | 12 | 19.9±8.4 | 0.120 | 0.379 | 6.7±3.2 | 0.520 | 0.633 | 26.7±9.1 | **0.019** | 0.339 | 15.3±8.6 | 0.329 | 0.619 |
| >100 | 19 | 17.6±7.2 |  |  | 6.1±2.4 |  |  | 14.7±8.7 |  |  | 11.0±6.9 |  |  |

**Table S4. Comparison of NK cell activity with pathologic parameters related to tumor progression in patients undergoing pancreatic surgery (Resectable group, n = 16).**

| Factors | No. of patient | K562 stimulation | | | | | | P815-ULBP1+CD48 stimulation | | | | | |
| --- | --- | --- | --- | --- | --- | --- | --- | --- | --- | --- | --- | --- | --- |
|  |  | CD107a+ (%) | | | IFN-γ+ (%) | | | CD107a+ (%) | | | IFN-γ+ (%) | | |
|  |  | Mean ± SD | *P*-value | | Mean ± SD | *P*-value | | Mean ± SD | *P*-value | | Mean ± SD | *P*-value | |
|  |  |  | Univariate | Multivariate |  | Univariate | Multivariate |  | Univariate | Multivariate |  | Univariate | Multivariate |
| Tumor location | | | | | | | | | | | | | |
| Head/neck | 7 | 20.3±8.5 | 0.721 | 0.761 | 7.3±2.7 | 0.789 | 0.488 | 2.1±10.1 | 0.903 | 0.503 | 8.3±5.5 | 0.184 | 0.070 |
| Body/tail | 9 | 16.0±11.5 |  |  | 4.9±3.3 |  |  | 23.4±10.5 |  |  | 15.0±5.7 |  |  |
| Tumor size | | | | | | | | | | | | | |
| <2cm | 5 | 26.0±9.8 | 0.544 | 0.446 | 4.2±2.0 | 0.520 | 0.294 | 27.1±5.9 | 0.286 | 0.414 | 15.2±4.2 | 0.607 | 0.633 |
| >2cm | 11 | 18.5±10.3 |  |  | 7.0±3.3 |  |  | 16.6±11.2 |  |  | 13.0±6.6 |  |  |
| Degree of tumor differentiation | | | | | | | | | | | | | |
| Wel/Mod | 10 | 23.2±9.5 | **0.046** | 0.079 | 7.9±2.9 | 0.099 | 0.102 | 27.9±9.1 | **0.016** | **0.043** | 13.7±5.5 | 0.230 | 0.172 |
| Poor | 5 | 11.0±4.9 |  |  | 3.7±2.4 |  |  | 10.6±4.8 |  |  | 8.8±6.9 |  |  |
| NA | 1 |  |  |  |  |  |  |  |  |  |  |  |  |
| Perineural invasion | | | | | | | | | | | | | |
| Absent | 5 | 20.2±10.4 | 0.952 | 0.919 | 4.9±3.1 | 0.406 | 0.654 | 27.8±11.2 | 0.642 | 0.795 | 15.6±6.9 | 0.775 | 0.641 |
| Present | 11 | 19.4±10.3 |  |  | 6.8±2.9 |  |  | 19.1±9.9 |  |  | 11.3±5.8 |  |  |
| Lymphovascular invasion | | | | | | | | | | | | | |
| Absent | 7 | 19.4±10.2 | 0.787 | 0.246 | 7.3±2.1 | 0.343 | 0.546 | 27.9±10.0 | 0.517 | 0.739 | 15.2±3.6 | 0.224 | 0.312 |
| Present | 9 | 19.0±10.4 |  |  | 3.7±3.5 |  |  | 18.6±10.3 |  |  | 8.7±7.0 |  |  |
| Lymph node metastasis | | | | | | | | | | | | | |
| 0 | 4 | 26.0±8.7 | 0.662 | 0.571 | 5.5±2.2 | 0.852 | 0.645 | 27.9±3.9 | 0.217 | 0.331 | 16.0±1.2 | 0.356 | 0.605 |
| 0-2 | 6 | 16.4±11.2 |  |  | 4.0±2.7 |  |  | 21.1±9.5 |  |  | 8.7±6.5 |  |  |
| >3 | 6 | 19.0±10.2 |  |  | 7.4±3.8 |  |  | 16.6±11.7 |  |  | 13.0±6.3 |  |  |

Wel; well, Mod; moderate, NA; not available,

**Table S5. Comparison of the level of multiple cytokines among HCs, non-malignant group, and malignant group.**

| Sample ID | Cytokines (pg/ml) | | | | | | | | |
| --- | --- | --- | --- | --- | --- | --- | --- | --- | --- |
|  | TNF-α | TGF-β1 | TGF-β2 | TGF-β3 | IL-4 | IL-5 | IL-6 | IL-10 | IL-13 |
| HC_1 | <8.88 | 4219.73 | <22.54 | <42.55 | <9.84 | <5.76 | 1.06 | <3.59 | <454.81 |
| HC_2 | <8.88 | 8239.01 | <22.54 | <42.55 | <9.84 | <5.76 | 0.80 | <3.59 | <454.81 |
| HC_3 | <8.88 | 3183.53 | <22.54 | <42.55 | <9.84 | <5.76 | 1.06 | <3.59 | <454.81 |
| HC_4 | <8.88 | 3907.56 | <22.54 | <42.55 | <9.84 | <5.76 | 1.20 | <3.59 | <454.81 |
| HC_5 | <8.88 | 9844.99 | <22.54 | <42.55 | <9.84 | <5.76 | 1.20 | <3.59 | <454.81 |
| HC_6 | <8.88 | 11874.42 | <22.54 | <42.55 | <9.84 | <5.76 | 1.20 | <3.59 | <454.81 |
| HC_7 | <8.88 | 7037.18 | <22.54 | <42.55 | <9.84 | <5.76 | 1.59 | <3.59 | <454.81 |
| HC_8 | <8.88 | 7729.63 | <22.54 | <42.55 | <9.84 | <5.76 | 1.33 | <3.59 | <454.81 |
| HC_9 | <8.88 | 7067.37 | <22.54 | <42.55 | <9.84 | <5.76 | 1.06 | <3.59 | <454.81 |
| HC_10 | <8.88 | 9163.07 | <22.54 | <42.55 | <9.84 | <5.76 | 2.93 | <3.59 | <454.81 |
| HC_11 | <8.88 | 5578.03 | <22.54 | <42.55 | <9.84 | <5.76 | 1.06 | <3.59 | <454.81 |
| HC_12 | <8.88 | 7006.98 | <22.54 | <42.55 | <9.84 | <5.76 | 1.06 | <3.59 | <454.81 |
| HC_13 | <8.88 | 7639.53 | <22.54 | <42.55 | <9.84 | <5.76 | 1.20 | <3.59 | <454.81 |
| HC_14 | <8.88 | 7609.48 | <22.54 | <42.55 | <9.84 | <5.76 | 1.06 | <3.59 | <454.81 |
| HC_15 | <8.88 | 5761.59 | <22.54 | <42.55 | <9.84 | <5.76 | 0.80 | <3.59 | <454.81 |
| HC_16 | <8.88 | 12166.71 | <22.54 | <42.55 | <9.84 | <5.76 | 0.80 | <3.59 | <454.81 |
| HC_17 | <8.88 | 21171.16 | <22.54 | <42.55 | <9.84 | <5.76 | 1.20 | <3.59 | <454.81 |
| HC_18 | <8.88 | 19784.34 | <22.54 | <42.55 | <9.84 | <5.76 | 1.06 | <3.59 | <454.81 |
| Non-Malignant_1 | <8.88 | 5914.28 | <22.54 | <42.55 | <9.84 | <5.76 | 4.01 | <3.59 | <454.81 |
| Non-Malignant_2 | <8.88 | 10641.88 | <22.54 | <42.55 | <9.84 | <5.76 | 2.93 | <3.59 | <454.81 |
| Non-Malignant_3 | <8.88 | 8059.46 | <22.54 | <42.55 | <9.84 | <5.76 | 1.06 | <3.59 | <454.81 |
| Non-Malignant_4 | <8.88 | 5883.76 | <22.54 | <42.55 | <9.84 | <5.76 | 2.79 | <3.59 | <454.81 |
| Non-Malignant_5 | <8.88 | 4032.60 | <22.54 | <42.55 | <9.84 | <5.76 | 2.39 | <3.59 | <454.81 |
| Non-Malignant_6 | <8.88 | 4032.60 | <22.54 | <42.55 | <9.84 | <5.76 | 1.59 | <3.59 | <454.81 |
| Non-Malignant_7 | <8.88 | 8328.70 | <22.54 | <42.55 | <9.84 | <5.76 | 1.33 | <3.59 | <454.81 |
| Non-Malignant_8 | <8.88 | 3813.62 | <22.54 | <42.55 | <9.84 | <5.76 | 1.33 | <3.59 | <454.81 |
| Non-Malignant_9 | <8.88 | 6795.37 | <22.54 | <42.55 | <9.84 | <5.76 | 1.06 | <3.59 | <454.81 |
| Non-Malignant_10 | <8.88 | 4219.73 | <22.54 | <42.55 | <9.84 | <5.76 | 1.06 | <3.59 | <454.81 |
| Non-Malignant_11 | <8.88 | 7729.63 | <22.54 | <42.55 | <9.84 | <5.76 | 1.33 | <3.59 | <454.81 |
| Non-Malignant_12 | <8.88 | 9963.29 | <22.54 | <42.55 | <9.84 | <5.76 | 0.80 | <3.59 | <454.81 |
| Non-Malignant_13 | <8.88 | 3782.27 | <22.54 | <42.55 | <9.84 | <5.76 | 2.12 | <3.59 | <454.81 |
| Non-Malignant_14 | <8.88 | 7097.55 | <22.54 | <42.55 | <9.84 | <5.76 | 1.33 | <3.59 | <454.81 |
| Non-Malignant_15 | <8.88 | 3341.71 | <22.54 | <42.55 | <9.84 | <5.76 | 1.86 | <3.59 | <454.81 |
| Non-Malignant_16 | <8.88 | 5669.85 | <22.54 | <42.55 | <9.84 | <5.76 | 3.20 | <3.59 | <454.81 |
| Non-Malignant_17 | <8.88 | 11552.40 | <22.54 | <42.55 | <9.84 | <5.76 | 1.86 | <3.59 | <454.81 |
| Non-Malignant_18 | <8.88 | 7278.48 | <22.54 | <42.55 | <9.84 | <5.76 | 1.86 | <3.59 | <454.81 |
| Non-Malignant_19 | <8.88 | 4716.35 | <22.54 | <42.55 | <9.84 | <5.76 | 1.06 | <3.59 | <454.81 |
| Malignant_1 | <8.88 | 12982.90 | <22.54 | <42.55 | <9.84 | <5.76 | 2.93 | <3.59 | <454.81 |
| Malignant_2 | <8.88 | 28696.52 | <22.54 | <42.55 | <9.84 | <5.76 | 8.27 | <3.59 | <454.81 |
| Malignant_3 | <8.88 | 33142.32 | <22.54 | <42.55 | <9.84 | <5.76 | 2.12 | <3.59 | <454.81 |
| Malignant_4 | <8.88 | 35995.19 | <22.54 | <42.55 | <9.84 | <5.76 | 20.98 | <3.59 | <454.81 |
| Malignant_5 | <8.88 | 16075.05 | <22.54 | <42.55 | <9.84 | <5.76 | 3.74 | <3.59 | <454.81 |
| Malignant_6 | <8.88 | 32509.48 | <22.54 | <42.55 | <9.84 | <5.76 | 1.33 | <3.59 | <454.81 |
| Malignant_7 | <8.88 | 5363.42 | <22.54 | <42.55 | <9.84 | <5.76 | 1.59 | <3.59 | <454.81 |
| Malignant_8 | <8.88 | 16734.78 | <22.54 | <42.55 | <9.84 | <5.76 | 0.80 | <3.59 | <454.81 |
| Malignant_9 | <8.88 | 2833.81 | <22.54 | <42.55 | <9.84 | <5.76 | 2.12 | <3.59 | <454.81 |
| Malignant_10 | <8.88 | 28141.49 | <22.54 | <42.55 | <9.84 | <5.76 | 6.20 | <3.59 | <454.81 |
| Malignant_11 | <8.88 | 23592.22 | <22.54 | <42.55 | <9.84 | <5.76 | 3.20 | <3.59 | <454.81 |
| Malignant_12 | <8.88 | 18932.39 | <22.54 | <42.55 | <9.84 | <5.76 | 1.06 | <3.59 | <454.81 |
| Malignant_13 | <8.88 | 22890.04 | <22.54 | <42.55 | <9.84 | <5.76 | 2.52 | <3.59 | <454.81 |
| Malignant_14 | <8.88 | 28308.07 | <22.54 | <42.55 | <9.84 | <5.76 | 4.28 | <3.59 | <454.81 |
| Malignant_15 | <8.88 | 12020.62 | <22.54 | <42.55 | <9.84 | <5.76 | 3.60 | <3.59 | <454.81 |
| Malignant_16 | <8.88 | 41852.51 | <22.54 | <42.55 | <9.84 | <5.76 | 1.59 | <3.59 | <454.81 |
| Malignant_17 | <8.88 | 9637.76 | <22.54 | <42.55 | <9.84 | <5.76 | 0.80 | <3.59 | <454.81 |
| Malignant_18 | <8.88 | 8806.07 | <22.54 | <42.55 | <9.84 | <5.76 | 1.06 | <3.59 | <454.81 |
| Malignant_19 | <8.88 | 26583.91 | <22.54 | <42.55 | <9.84 | <5.76 | 1.59 | <3.59 | <454.81 |
| Malignant_20 | <8.88 | 6370.94 | <22.54 | <42.55 | <9.84 | <5.76 | 1.59 | <3.59 | <454.81 |
| Malignant_21 | <8.88 | 17878.43 | <22.54 | <42.55 | <9.84 | <5.76 | 1.59 | <3.59 | <454.81 |
| Malignant_22 | <8.88 | 7278.48 | <22.54 | <42.55 | <9.84 | <5.76 | 119.28 | <3.59 | <454.81 |
| Malignant_23 | <8.88 | 5914.28 | <22.54 | <42.55 | <9.84 | <5.76 | 0.93 | <3.59 | <454.81 |
| Malignant_24 | <8.88 | 4716.35 | <22.54 | <42.55 | <9.84 | <5.76 | 3.20 | <3.59 | <454.81 |
| Malignant_25 | <8.88 | 6188.52 | <22.54 | <42.55 | <9.84 | <5.76 | 1.86 | <3.59 | <454.81 |
| Malignant_26 | <8.88 | 3625.31 | <22.54 | <42.55 | <9.84 | <5.76 | 91.95 | <3.59 | <454.81 |
